# Electronic Structure of Metalloporphenes, Antiaromatic Analogues of Graphene

**DOI:** 10.1021/jacs.3c12079

**Published:** 2024-01-31

**Authors:** Ivan Pavlak, Lujo Matasović, Eric A. Buchanan, Josef Michl, Igor Rončević

**Affiliations:** † Department of Chemistry, Faculty of Science, University of Zagreb, Horvatovac 102A, Zagreb 10000, Croatia; ‡ Cavendish Laboratory, Department of Physics, University of Cambridge, J. J. Thomson Avenue, Cambridge CB3 0HE, U.K.; § Department of Chemistry and Biochemistry, University of Colorado, Boulder, Colorado 80309-0215, United States; ∥ Institute of Organic Chemistry and Biochemistry of the CAS, Flemingovo nám. 2, Prague 6 16610, Czech Republic; ⊥ Department of Chemistry, University of Oxford, Chemistry Research Laboratory, Oxford OX1 3TA, U.K.

## Abstract

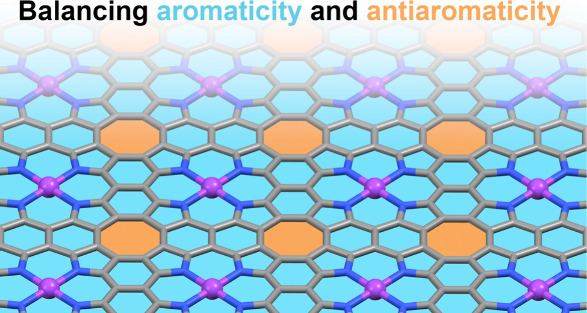

Zinc porphene is a two-dimensional material made of fully fused zinc porphyrins in a tetragonal lattice. It has a fully conjugated π-system, making it similar to graphene. Zinc porphene has recently been synthesized, and a combination of rough conductivity measurements and infrared and Raman spectroscopies all suggested that it is a semiconductor (Magnera, T.F. et al. Porphene and Porphite as Porphyrin Analogs of Graphene and Graphite, *Nat. Commun.***2023**, 14, 6308). This is in contrast with all previous predictions of its electronic structure, which indicated metallic conductivity. We show that the gap-opening in zinc porphene is caused by a Peierls distortion of its unit cell from square to rectangular, thus giving the first account of its electronic structure in agreement with the experiment. Accounting for this distortion requires proper treatment of electron delocalization, which can be done using hybrid functionals with a substantial amount of exact exchange. Such a functional, PBE38, is then applied to predict the properties of many first transition row metalloporphenes, some of which have already been prepared. We find that changing the metal strongly affects the electronic structure of metalloporphenes, resulting in a rich variety of both metallic conductors and semiconductors, which may be of great interest to molecular electronics and spintronics. Properties of these materials are mostly governed by the extent of the Peierls distortion and the number of electrons in their π-system, analogous to changes in aromaticity observed in cyclic conjugated molecules upon oxidation or reduction. These results give an account of how the concept of antiaromaticity can be extended to periodic systems.

## Introduction

Two-dimensional (2D) polymers have been extensively investigated due to their promising applications as optoelectronic materials, molecular magnets, energy storage media, electrocatalysts, etc.^1−8^ The archetypal 2D material is graphene, which displays remarkable mechanical and electronic properties due to a fully conjugated π-system.^9,10^ However, stoichiometric functionalization of graphene remains a towering obstacle in the path toward tunable π-conjugated 2D materials.^11,12^

**Figure 1:**
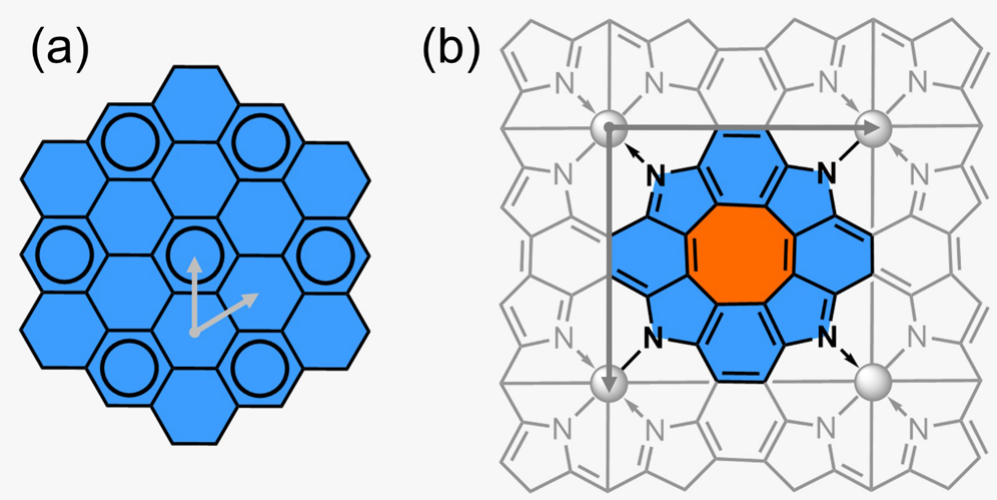
Structure of (a) graphene and (b) metalloporphene (MP), with lattice vectors shown with gray arrows. Aromatic and antiaromatic rings are shown in blue and orange, respectively.

Recently, we have succeeded in the synthesis of porphene, a graphene analogue made of fully fused porphyrin rings (Figure 1).^13^ Both graphene and porphene are fully conjugated, but they have two key differences. One, graphene has a hexagonal lattice, while porphene is composed of roughly square porphyrin monomers. A half-filled square lattice is known to be susceptible to distortion,^14^ suggesting that the shape of the porphene unit cell may depend on the number of electrons in its π-system. To draw a parallel to molecules, graphene can be thought of as a periodic analogue of the aromatic high-symmetry benzene,^15−17^ while porphene resembles antiaromatic cyclobutadiene, which adopts a lower-symmetry rectangular geometry. This was first noted by Osuka and collaborators on a 2 × 2 fragment of zinc porphene (Figure 1b).^18^

The second, more practically important difference between graphene and porphene is that the latter offers a straightforward avenue for tuning. Different metalloporphenes (MP) can be produced from free-base porphene by controlled reversible insertion of a metal ion, M, into the center of each macrocycle, without taking any π-centers out of conjugation.^14^ Given the wide choice of metals M in different spin states and possible axial ligands X, porphene offers an unprecedented opportunity for stoichiometric functionalization. Availability of bidentate ligands suggests that controlled stacking into periodic multilayers may be within reach. Since metal ions can be introduced into porphene and removed under controlled conditions, nanolithographic writing of patterns and design of circuitry for applications in electronics, spintronics, photonics, and elsewhere can be imagined. The ultimate resolution of this canvas for molecular painting is 8.4 Å, the closest distance between two metal centers in a metalloporphene.

To take full advantage of the opportunities offered by this synthetic advance, it is important to obtain guidance from theory concerning metalloporphene properties as a function of the choice of metal and ligands. Such calculations have been published before,^19−24^ but they invariably predicted zinc porphene (**ZnP**) to be a square metallic conductor. This is in disagreement with our experimental results on **ZnP**, which indicated an absence of metallic conductivity and showed a presence of infrared-active vibrations, revealing that **ZnP** is in fact a semiconductor (nonzero band gap).^13^ In the Symmetry Breaking in **ZnP** section of this paper, we show that an appropriate level of theory can account for the observed semiconducting nature of **ZnP**, and in the First Transition Row Metalloporphenes section, we systematically investigate the electronic structure and geometry of metalloporphenes containing elements from the first transition row of the periodic table, Sc–Zn, revealing a rich landscape of tunable materials.

## Results and Discussion

### Symmetry Breaking in **ZnP**

Metalloporphenes such as **ZnP** are composed of both aromatic circuits (pyrrole and benzene, shown in blue in Figure 1) with delocalized π-bonds and antiaromatic circuits (cylooctatetraene, orange) with distinctly localized bonds.^18^ This leads to mixed aromaticity (or concealed antiaromaticity^25^), suggesting that both types of circuits need to be described with a similar level of accuracy to properly capture their electronic structure. All previous investigations of **ZnP**(19−22) used pure density functional theory (DFT) to optimize its geometry, predicting a *D*_4h_ (square) unit cell with a single minimum. Subsequent band structure calculations on this square minimum always yielded a gapless band structure, in contrast with the experiment^13^ as well as with the observation that 2D materials with square lattices are exceedingly rare.^26,27^

This discrepancy between the theory and experiment may be explained by recognizing that pure DFT overemphasizes delocalization.^27^ In terms of the semiempirical Hubbard model,^28^ pure DFT underestimates *U* (the energy lost when two electrons occupy the same site) and overestimates the hopping integral *t* (the energy gained when an electron is free to move between sites). This leads to a too small *U*/*t* ratio, overemphasizing delocalization.^29^

Hybrid DFT can provide a better balance between localization (*U*) and delocalization (*t*) by including some percentage (usually 20–50%) of exact exchange (EE), which lowers the energy of all same-spin electron pairs. Therefore, the addition of EE promotes localization, or increases *U*/*t*, which comes at the expense of increased computation time and worse description of static correlation.^30^

**Figure 2:**
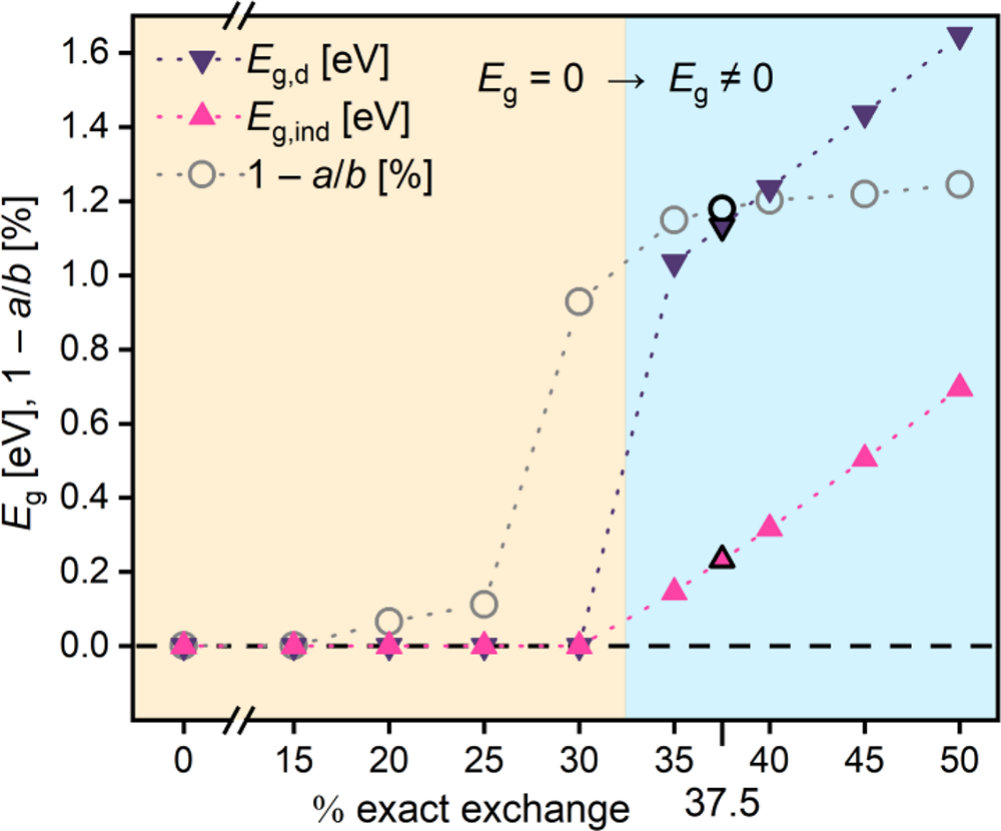
Direct (*E*_g,d_, purple triangles) and indirect band gap (*E*_g,ind_, pink triangles) and unit cell asymmetry (1 – *a*/*b*; gray circles) of **ZnP** at different levels of EE, obtained using PBE*xx*, where *xx* indicates the amount of exact exchange. Metallic conductors are shown in orange, and semiconductors are in blue. Results obtained using PBE38 are outlined in black.

By admixing different proportions of EE to the pure PBE (Perdew–Burke–Ernzerhof^31^) functional, we found that at least 35% EE is necessary to break the square symmetry and open a band gap in **ZnP** (Figure 2). Similar results were previously obtained in the case of cyclo[18]carbon (an 18-membered all-carbon ring), an *s*-indacene derivative, and butadiyne-linked Zn porphyrin nanorings, which all require 30–35% EE to optimize to the experimentally found symmetry-broken geometries (see the Functional Choice section in the Supporting Information).^32−34^ Additionally, around 35% of exchange is well established as the ideal proportion for distinguishing classes of mixed-valence systems^35,36^ and molecular wires^37−39^ with different degrees of electron delocalization. For these reasons, all subsequent work presented here was done using the PBE38 (37.5% exact exchange) functional, which was also found to be the optimal proportion of EE in several benchmarks.^40,41^

### **ZnP** and Antiaromatic Molecules

**Figure 3:**
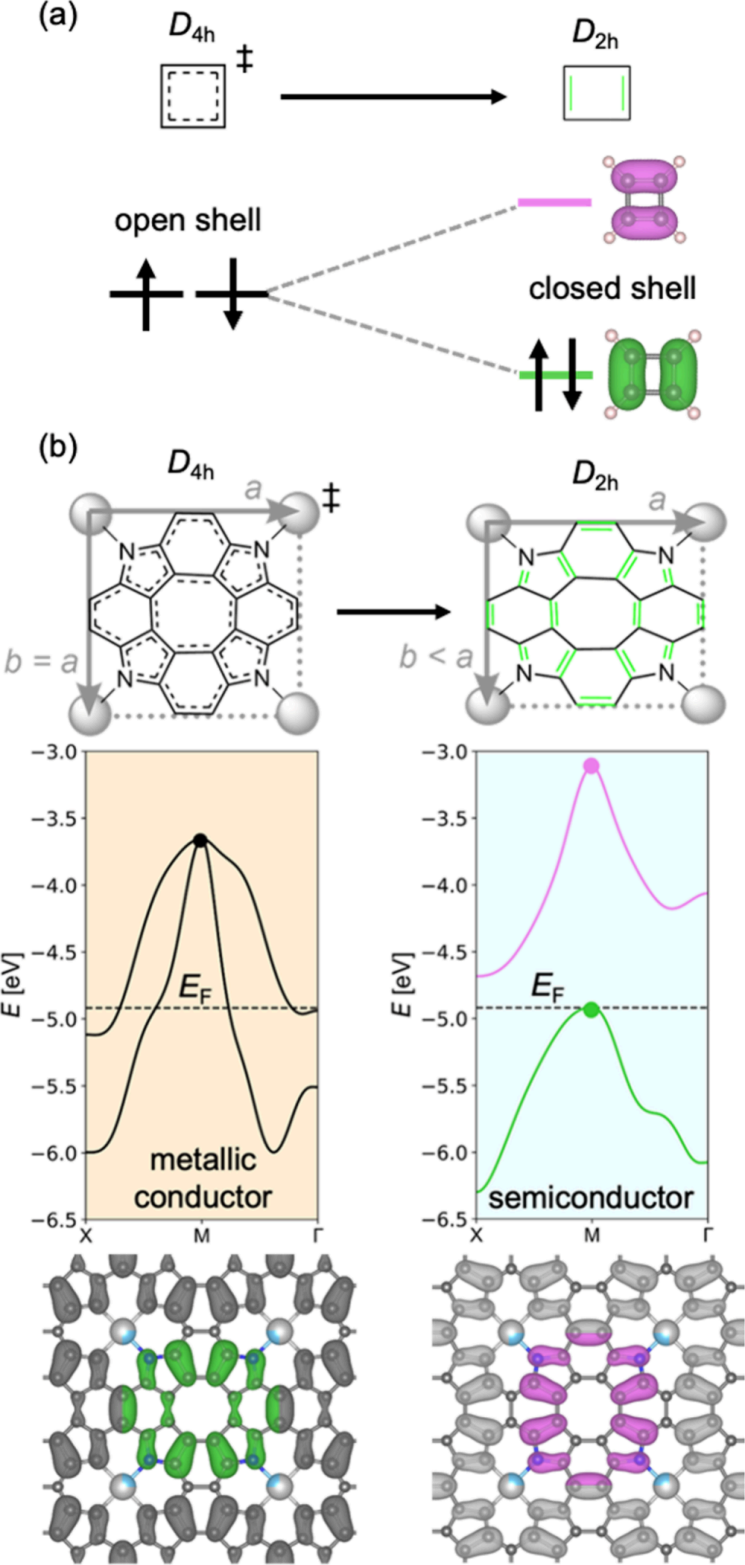
(a) Symmetry breaking in cyclobutadiene. (b) Peierls distortion in **ZnP**. Density of frontier bands in **ZnP** in *D*_2h_ geometry at the M reciprocal space point. Densities associated with the highest occupied and lowest unoccupied orbitals or bands are shown in green and pink, respectively.

The symmetry breaking and gap-opening in **ZnP** may be rationalized by comparing it to antiaromatic (4*n*) annulenes, where *n* is the number of carbon atoms. In the absence of bond length alternation (BLA), 4*n* annulenes such as cyclobutadiene (*n* = 1) or planar cyclooctatetraene (*n* = 2) have a half-filled degenerate orbital pair related by a 90° rotation (i.e., sine and cosine solutions of the Hückel model; Figure 3a). This *D*_4*n*h_ configuration is unstable; in fact, it is a transition structure connecting two equivalent broken symmetry *D*_2*n*h_ minima, which have nonzero BLA.^42,43^ Introducing BLA breaks the degeneracy of the orbital pair, with one orbital (with density on shorter bonds) becoming occupied (i.e., the HOMO) and the other (with density on longer bonds) being unoccupied (i.e., the LUMO; Figure 3a). As a result, the extent of BLA in 4*n* annulenes directly affects their HOMO–LUMO gap.

The band structure of *D*_4h_**ZnP** shows two half-filled bands related by a 90° rotation (Figure 3b). They are responsible for their metallic conductivity. At the M reciprocal space point, at which the wave function is periodic with respect to the square 2 × 2 porphene fragment, which contains the antiaromatic eight-membered circuit (whole structure in Figure 1b), these two bands are exactly degenerate. Breaking the *D*_4h_ symmetry by introducing BLA lowers the energy of the band concordant with the introduced BLA pattern (making more of it filled) and increases the energy of its rotational partner (making less of it filled), in a direct analogy to symmetry breaking in cyclobutadiene (Figure 3). When this Peierls distortion is sufficiently large, a metal–insulator transition occurs (Figure 3b, right). Therefore, **ZnP** is an antiaromatic 2D polymer, characterized by two equivalent semiconductive *D*_2h_ minima connected by a gapless *D*_4h_ transition structure (a 3 × 3 **ZnP** fragment shows similar behavior and properties; see Figure S4 in the Supporting Information).

### First Transition Row Metalloporphenes

To understand the effect of inserting different metals into porphenes, we have investigated 57 first-row metalloporphenes in their *D*_2h_ and *D*_4h_ minima. These metalloporphenes can be described with the general formula ^2S+1^M(*q*)XP, where the metal M, which is in the oxidation state *q*, ranges from Sc to Zn. X denotes the axial ligand (oxide or chloride, if present), and 2S + 1 is the unit cell multiplicity. In addition to band structures, we report the band gap *E*_g_, effective masses *m*_e_ (in semiconductors) and conductivity (in gapless systems), the energy difference between the lowest-energy *D*_2h_ and *D*_4h_ geometries (when they are distinct), and the work function. Finally, the variation in C–C bond lengths is described with a modified HOMA.^44,45^ Further details are given in the Methods section and the Supporting Information. We have also prepared an interactive web interface for viewing our results, available at https://metalloporphene-electronic-structure.onrender.com/.

**Chart 1:**
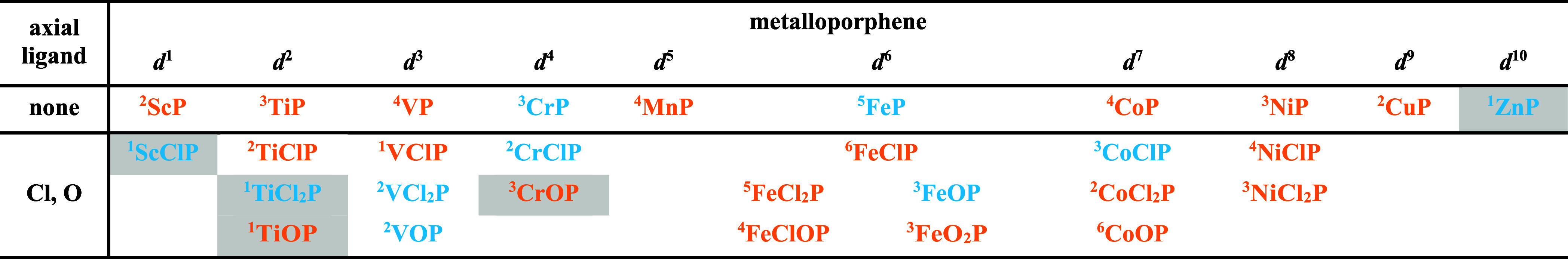
Most Stable Electronic States of First-Row Metalloporphenesa

In virtually all cases, we found that the bands close to the Fermi level have a dominant π-character (Figures 5 and 6), indicating that the analogy between metalloporphenes and annulenes may be generally valid. This motivated us to classify the metalloporphenes according to the number of π-electrons in their unit cell (Table 1). Chart 1 displays a “periodic table” of first transition row metalloporphenes, showcasing a variety of open- and closed-shell metallic conductors and semiconductors.

a Gapless metalloporphenes are shown in blue and semiconductors in orange; closed-shell singlets are shown with a gray background.

**Table 1:**
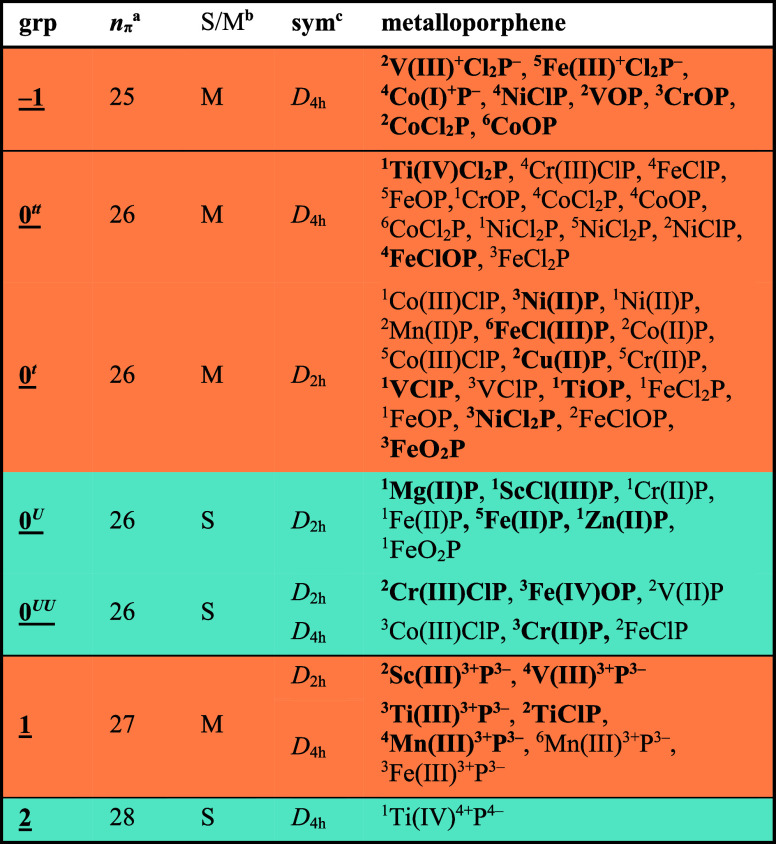
Classification of Metalloporphenes

a Bold: lowest-energy spin state for this combination of metal and ligand. Orange: metallic conductors; blue: semiconductors. Number of π-electrons in the unit cell.

b Presence (S) or absence (M) of a band gap.

c Point group of the lowest minimum.

**Figure 4:**
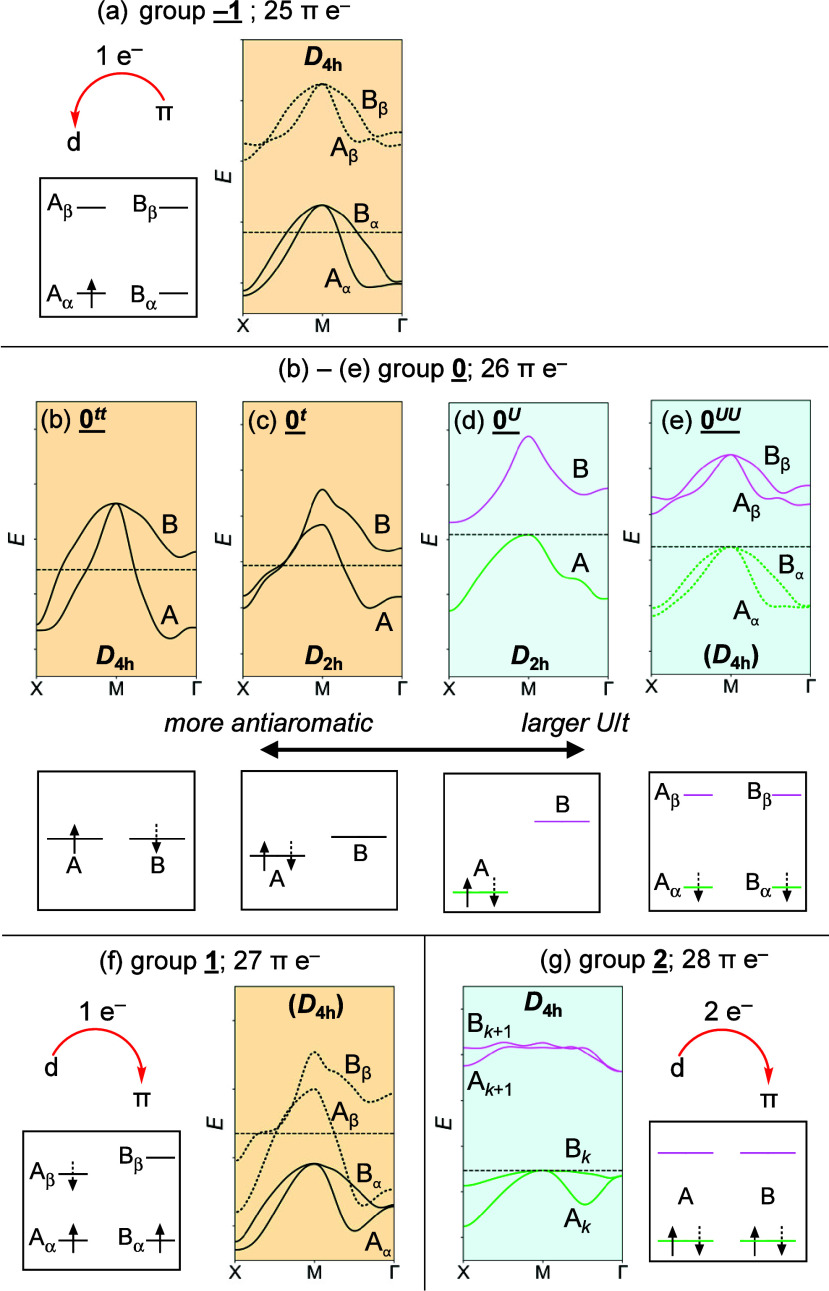
Minimal band structures and analogous MO diagrams for metalloporphenes in groups **–1** (a), **0***^**tt**^* (b), **0***^**t**^* (c), **0***^**U**^* (d), **0*****^UU^*** (e), **1** (f), and **2** (g), showing only bands close to the Fermi level along X-M-Γ and frontier molecular orbitals. In spin-polarized systems, dashed (full) lines and arrows correspond to α (β) spin.

Most metalloporphenes, including **Zn(II)P**, belong to group **0**, in which the number of π-electrons remains unperturbed by the presence of the metal. Therefore, all group **0** metalloporphenes have 26 π-electrons per unit cell and display antiaromatic features. They can be divided further according to the extent of electron delocalization or ascending *U*/*t* ratio: **0**^***tt***^ (no Peierls distortion, gapless), **0**^***t***^ (distorted, gapless), **0**^***U***^ (antiaromatic semiconductors), and **0**^***UU***^ (Baird aromatic semiconductors), as shown in Figure 4.

In group **–1** (25 π-electrons per unit cell), an electron is transferred from the π-system into a low-energy d orbital, while in group **1** (27 π e^–^), an electron is donated from a high-energy d orbital into the π-system. Members of both **–1** and **1** are half-metals, which may be compared to open-shell radical cations (group **–1**) or anions (**1**) of annulenes.

The only first transition row member of group **2** is singlet titanium porphene, in which titanium donates both of its d electrons to the π-system, resulting in 28 π-electrons per unit cell and a metalloporphene, which may be written as ^**1**^**Ti(IV)**^**4+**^**P**^**4–**^. Adding two electrons to conjugated hydrocarbons usually reverses their aromaticity,^46,47^ so it is not surprising that ^**1**^**Ti(IV)**^**4+**^**P**^**4–**^ shows behavior consistent with an aromatic compound.

To emphasize electron transfer, formulas of metalloporphenes in groups **–1**, **1**, and **2** are written as in the example above. In Figure 4, minimal band structures for each metalloporphene group are shown alongside analogous frontier MO diagrams. Throughout the text, metallic conductors are shown in orange and semiconductors in blue. The next section provides an overview of metalloporphene groups with a few examples.

### Group **0**

**Figure 5:**
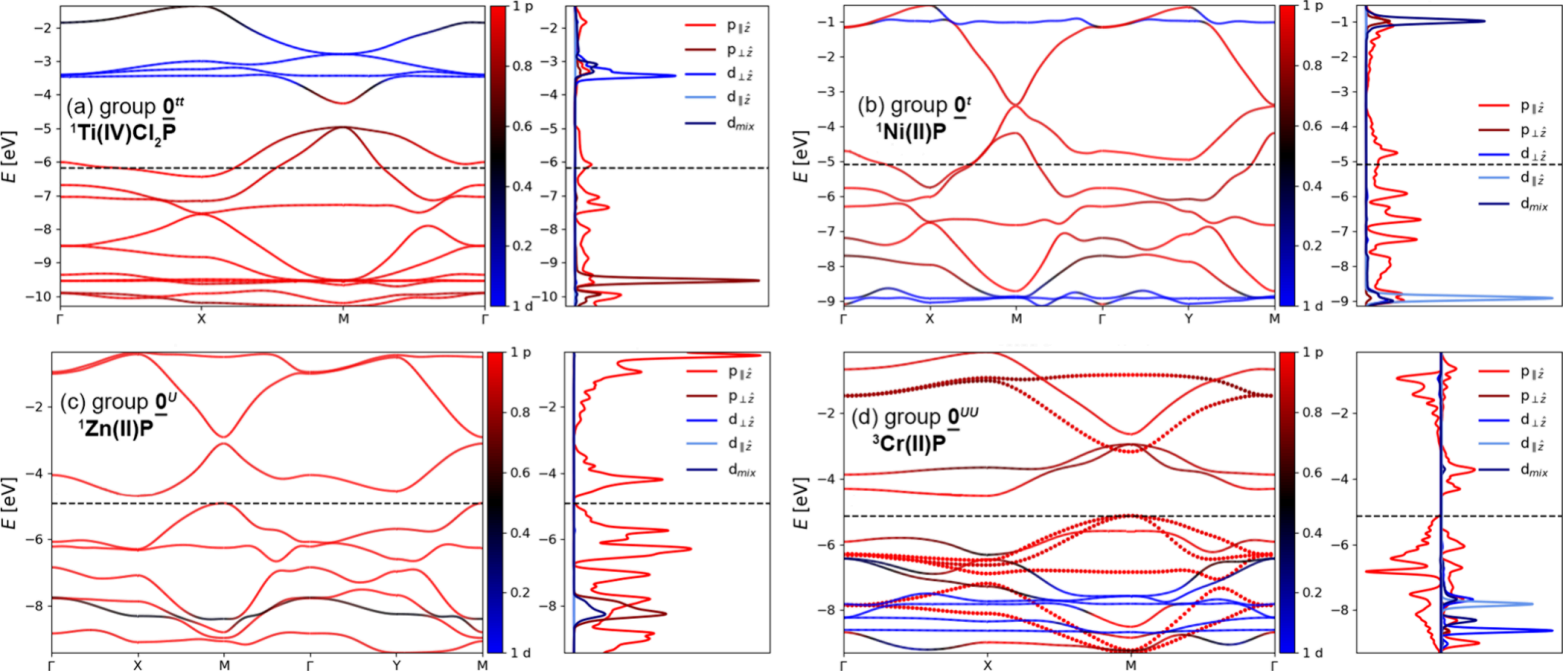
Band diagrams and density of state plots for (a) ^**1**^**Ti(IV)Cl**_**2**_**P** (**0^*tt*^**, *D*_4h_ geometry), (b) ^**1**^**Ni(II)P** (**0^*t*^**, *D*_2h_), (c) ^**1**^**Zn(II)P** (**0^*U*^**, *D*_2h_), and (d) ^**3**^**Cr(II)P** (**0^*UU*^**, *D*_2h_). Red and blue colors denote the p and d band character, respectively; a black dashed line shows the Fermi level (top of the valence band in semiconductors). In spin-polarized systems, different spins are shown with dotted and full lines in the band diagram and as positive and negative values in the density of state plots.

All metalloporphenes in group **0** have 26 π-electrons but differ significantly in their electronic structures and properties. Members of **0**^***tt***^ always feature axial ligands and have (mostly) empty d shells (e.g., ^**1**^**Ti(IV)Cl**_**2**_**P**; Figure 5a). They optimize to *D*_4h_ minima, which results in two half-filled bands degenerate at the M point (Figures 4b and 5a) and leads to metallic conductivity. These features are consistent with a small *U*/*t* ratio (*U* ≈ 0), which prevents the Peierls distortion and consequently the gap-opening (bond length variation is still present, cf. HOMA values in Table 2). Members of **0**^***tt***^ may be compared to *D*_4h_ cyclobutadiene (Figure 4b bottom), in which the rotationally related orbitals A and B share two opposite-spin electrons, leading to an open-shell singlet with very strong antiaromaticity.

**Table 2:** Properties of Selected Gapless Metalloporphenes

**grp**	**cmpd**	**Δ***E*^a^	***W***^b^	σ^c^	**HOMA**
**–1**	^**2**^**V(III)**^**+**^**Cl**_**2**_**P**^**–**^	0	6.17	0.33	0.62
	^**5**^**Fe(III)**^**+**^**Cl**_**2**_**P**^**–**^		6.58	0.84	0.39
	^**4**^**Co(I)**^**+**^**P**^**–**^		5.53	0.46	0.64
**0**^***tt***^	^**1**^**TiCl**_**2**_**P**	0	6.20	0.94	0.01
**0**^***t***^	^**6**^**FeClP**	0.03	5.57	0.49	0.41
	^**1**^**NiP**	0.01	5.08	0.91	0.40
	^**2**^**CuP**	0.05	4.90	0.47	0.39
**1**	^**2**^**Sc(III)**^**3+**^**P**^**3–**^	0.01	4.65	0.31	0.70
	^**3**^**Ti(III)**^**3+**^**P**^**3–**^	0.40	4.84	0.26	0.90
	^**4**^**V(III)**^**3+**^**P**^**3–**^	0.68	4.77	0.23	0.92
	^**4**^**Mn(III)**^**3+**^**P**^**3–**^	<0.01	4.93	0.29	0.89

a Energy difference between the *D*_4h_ and *D*_2h_ minima (eV).

b Work function (eV).

c Conductivity (MS/m).

Members of **0**^***t***^ are formed with metals that have at least a half-filled d shell (Mn–Cu). They undergo a Peierls distortion to *D*_2h_, which breaks the band degeneracy at the M point (Figure 4c) but is insufficiently large to open a band gap. This may be attributed to a slightly larger *U*/*t* ratio compared to **0**^***tt***^, resulting in a marginally more localized electronic structure. An analogous annulene would be cyclobutadiene with a very slight BLA (Figure 4c, bottom), in which both open-shell character and antiaromaticity are slightly reduced relative to **0**^***tt***^. Compared to other metalloporphenes, members of **0**^***tt***^ and **0**^***t***^ tend to have higher work functions and conductivities (Table 2).

^**1**^**Zn(II)P** (Figure 5c) belongs to the **0**^***U***^ group, in which electron repulsion is sufficiently large to both induce a distortion and open a band gap. This corresponds to a closed-shell electronic structure in cyclobutadiene at its equilibrium geometry (Figure 4d). The relatively high *U*/*t* ratio in **0**^***U***^ is reflected in large effective masses (corresponding to poorer electron mobility; Table 3), compared to similar porphyrin-based systems.^48^

**Table 3:** Properties of Selected Semiconductive Metalloporphenes

**grp**	**cmpd**	***E*_g,i_**^a^/*E*_g,d_	Δ*E*^b^	*W*^c^	***m*_VB_**^d^*/m*_CB_	**HOMA**
**0**^***U***^	^**1**^**ScClP**	0.31	0.06	5.77	0.10	0.02
		1.21			0.37	
	^**5**^**FeP**	0.48	0.43	4.89	0.17	0.09
		3.07			0.23	
	^**1**^**ZnP**	0.22	0.10	4.91	0.18	0.04
		1.13			0.41	
**0**^***UU***^	^**2**^**CrClP**	0.77	2.78	5.88	0.24	0.64
		2.22			0.16	
	^**3**^**CrP**	0.61	0	5.13	0.22	0.66
		2.12			0.15	
	^**3**^**CoClP**	0.82	0	5.68	0.25	0.59
		2.58			0.20	
**2**	^**1**^**Ti(VI)**^**4+**^**P**^**4–**^	2.17	0	5.54	1.20	0.74
		2.28			0.42	

a Indirect (*E*_g,i_) and direct (*E*_g,d_) band gap.

b Energy difference between the *D*_4h_ and D_2h_ minima (eV).

c Work function (eV).

d Valence (*m*_VB_) and conduction (*m*_CB_) eff. mass.

In the lowest triplet state of cyclobutadiene, both rotationally related orbitals A and B are singly occupied with the same-spin electrons, resulting in a Baird aromatic *D*_4h_ geometry (Figure 4e, bottom). If *U* is sufficiently large, then the triplet can even become the ground state, as found in some indenofluorenes.^49,50^ This is analogous to the electronic structure of the **0**^***UU***^ metalloporphenes (Figure 4e), which must be spin-polarized. As the frontier bands in **0**^***U***^ are spatially almost identical, these metalloporphenes have similar electron and hole mobilities. Due to their Baird aromatic character, they tend to have significantly larger band gaps and smaller BLA than **0**^***U***^ metalloporphenes (Table 3).

#### Groups **–1** and **1**

**Figure 6:**
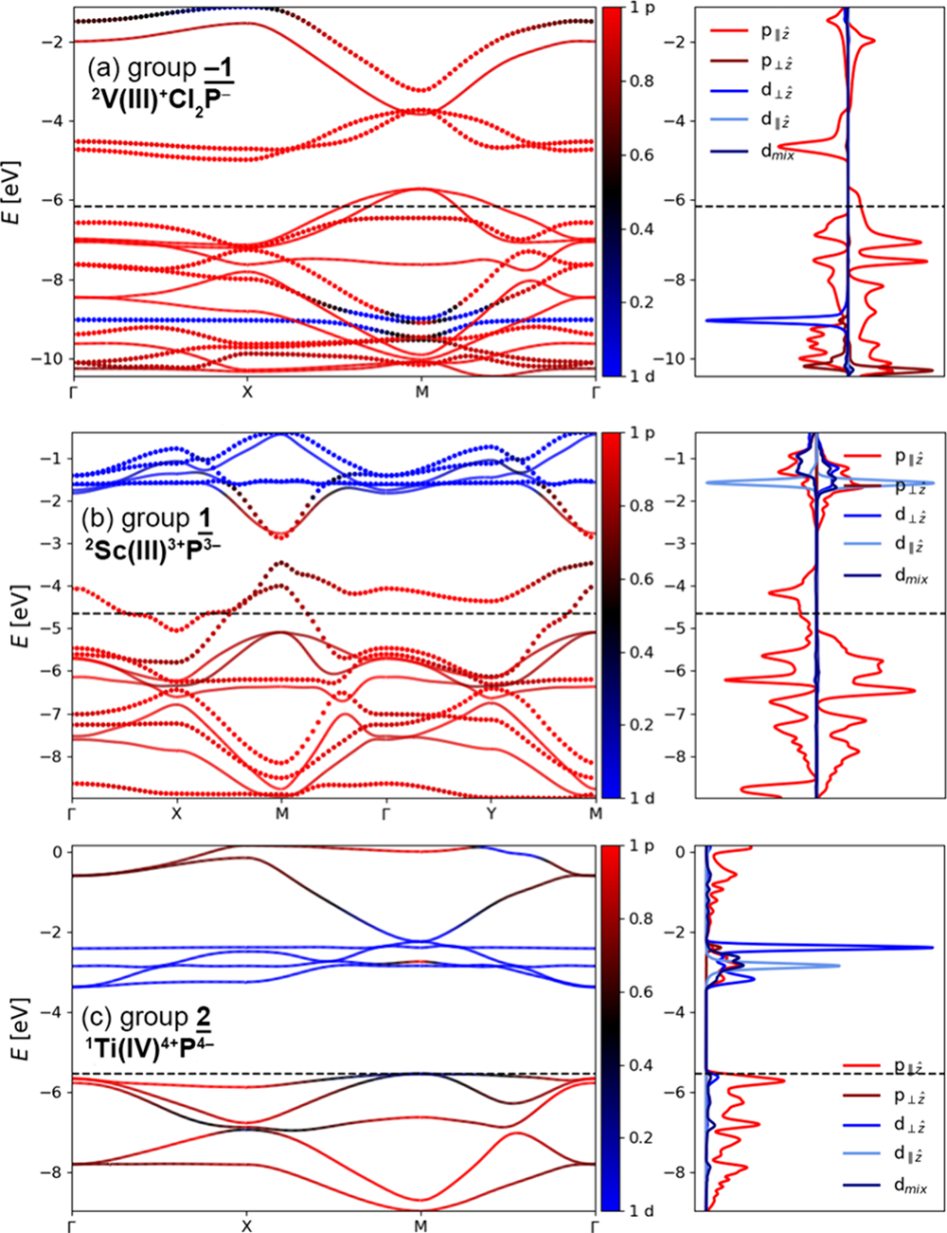
Band diagrams and density of state plots for (a) **^2^V(III)^+^Cl_2_P^–^** (group **–1**, *D*_4h_ geometry), (b) **^2^Sc(III)^3+^P^3–^** (**1**, *D*_2h_), and (c) **^1^Ti(IV)^4+^P^4–^**(**2**, *D*_4h_). Red and blue colors denote the p and d band character, respectively; the black dashed line shows the Fermi level (top of the valence band in semiconductors). In spin-polarized systems, different spins are shown with dotted and full lines in the band diagram and as positive and negative values in the density of state plots.

When the transition metal inserted into porphene has a vacant low-lying d orbital, an electron can be transferred from the π- to the d system, resulting in a group **–1** metalloporphene (Figure 4a). One such example of this is cobalt porphene in its quartet state (Figure 6a). If we assume that cobalt is divalent, then it will have a singly occupied low-energy d_*xy*_ orbital, which can accept an π-electron from porphene, thus making **^4^Co(I)^+^P^–^**.

Conversely, when the transition metal has an occupied high-energy d orbital, d → π single-electron transfer can occur, forming a group **1** metalloporphene. An obvious example is scandium porphene: in the absence of axial ligands, scandium will oxidize to Sc(III) by giving its electron to the π-system to form ^**2**^**Sc(III)**^**3+**^**P**^**–**^ (Figure 6b).

These metalloporphenes may be compared to radical cations and anions of annulenes, which tend to have low BLA.^51,52^ This leads to a small SOMO–SUMO gap, consistent with half-metallic behavior in groups **1** and **–1**.

### Group 2

Adding two electrons to the porphene π-system (relative to group **0**) completely fills up both rotationally related bands (Figure 4g), which leads to a *D*_4h_ geometry and a relatively large band gap (>2 eV; Table 3) in ^**1**^**Ti(IV)**^**4+**^**P**^**4–**^ (Figure 6c). Its electronic structure is similar to that of benzene (Figure 4f), indicating that a general form of Hückel’s rule (adding or removing two electrons reverses aromaticity) is applicable to the electronic structure of metalloporphenes. Group **2** metalloporphenes seem to be relatively rare, probably because they require metals with very high-energy d electrons (i.e., strong reducing agents).

### Outlook

At this point, it may be useful to identify several potential areas of application for metalloporphenes:

#### Molecular Wires

Due to their high conductivities, group **0**^***tt***^ and **0**^***t***^ metalloporphenes may be useful as molecular wires. Possible diamagnetic candidates are ^**1**^**Ti(IV)Cl**_**2**_**P** and ^**1**^**VClP**. Preparing these metalloporphenes would also provide a simple test of the predictions made here (see the Limitations section below).

#### Tunable Semiconductors

Semiconductive metalloporphenes (**0**^***U***^, **0**^***UU***^, and **2**) have a large variety of band gaps (1–3 eV), making them similar to quantum dots,^53^ except that the electronic properties are controlled by choice of metal instead of by size. Accurate band gap measurements (e.g., for ^**1**^**Zn(II)P**) will also enable the validation and refinement of these computational predictions.

#### Mechanical Properties

In **0**^***t***^ and **0**^***U***^ metalloporphenes, there are two equivalent *D*_2h_ orientations of each unit cell. As the barrier for their interconversion is low (∼0.1 eV/unit cell, cf. Tables 2 and 3 and the Supporting Information), the macroscopic properties of these metalloporphenes may be governed by statistics at the macroscopic scale and controlled by mechanical stretching.

#### Patterning

As noted previously,^13^ metalloporphenes might be used to build nanoscale circuits by inserting different metals in a specific pattern. Our results suggest that **0**^***UU***^ or **0**^***U***^ metalloporphenes could be used as a nonconductive blank canvas, which one could pattern by inserting metals forming **0**^***tt***^ and **0**^***t***^ metalloporphenes.

#### Magnetism

The potential for use of metalloporphenes in spintronics is currently speculative as their magnetic ground state and exchange coupling are unknown. Unless the exchange interaction through the π-system is very strong, the relatively large separation (∼8.5 Å) between metallic centers will prevent fast decoherence,^54^ which is essential for building multiqubit devices. Fine control over the strength of the exchange coupling could be achieved by metal choice: for example, metallic conductor ^**2**^**Cu(II)P** will likely have a larger coupling than semiconducting ^**2**^**Cr(III)ClP**. Finally, the half-metallic behavior (i.e., conduction through only a single spin channel) in groups **–1** (e.g., ^**2**^**V(III)**^**+**^**Cl**_**2**_**P**^**–**^) and **1** suggests applications in spin filtering.^55,56^

### Limitations

To paraphrase Lakatos, the quality of a theory is determined by the accuracy of its predictions.^57^ While hybrid DFT has undoubtedly been very successful in predicting properties of many materials, the procedure for choosing the ideal amount of exact exchange (or the Hubbard *U*) is not always clear, especially when geometry optimizations and large unit cells are involved, preventing the use of higher-level methods such as MP2 and GW. In the case of metalloporphenes, using less than 35% EE yields a qualitatively wrong electronic structure (Figure 2) while using a significantly larger amount overestimates localization, predicting too high spin multiplicities (e.g., PBE50 wrongly predicts the ground state of iron(II) porphyrin to be a quintet, while PBE0 and PBE38 correctly predict a triplet^58^).

To gauge the sensitivity of our results to the proportion of EE, we recomputed a subset (26 systems) of band structures at PBE0 (25% EE) and PBE50 (50% EE). Our results (Table S1 in the Supporting Information) show that metalloporphenes belonging to groups **0**^***t***^ and **0**^***U***^ are particularly sensitive to the amount of exchange, while the electronic structure of metalloporphenes in other groups is much less perturbed, thus increasing confidence in the majority of our results. Neglect of spin–orbit coupling may be a larger issue, but its inclusion is very costly due to the loss of time-reversal symmetry, making it impractical to perform such calculations on a large scale.

Finally, the properties of any metalloporphene sample will be strongly affected by defects present in the material. Due to the large size of the unit cell, we limited ourselves to investigating defects in a 2 × 2 **ZnP** supercell. This means that our results are much more extreme (25% porphyrins carry a defect) than anything that might be found in a somewhat ordered 2D material. Investigating the effects of (a) demetalation and double protonation of a (b) meso–meso or (c) β–β bond, we found that all three defects lower the band gap but that the system remains a semiconductor (see the Defects section in the Supporting Information).

Taking these limitations into account, we may suggest the synthesis of ^**1**^**Ti(IV)Cl**_**2**_**P** (group **0**^***tt***^) as a gapless metallic conductor (no ambiguity about the electronic state, little spin–orbit coupling, and gapless at least up to 50% EE), with ^**3**^**Ni(II)P** (**0**^***t***^) as a more experimentally accessible alternative^59^ (gapless at least up to 50% EE, ^**1**^**Ni(II)P** higher by 0.24 eV/macrocycle, and also gapless). We are similarly confident in the semiconductor nature (down to at least 25% EE) of aromatic ^**1**^**Ti(IV)**^**2+**^**P**^**4–**^ (group **2**), although ^**3**^**Cr(II)P** (**0**^***UU***^, quintet higher by 0.36 eV/macrocycle) might be easier to prepare.

## Conclusions

This paper provides a theoretical description of **ZnP** that agrees with experiments and accounts for its semiconductive nature. **ZnP** has an electronic structure analogous to that of antiaromatic molecules, making it susceptible to Peierls distortion. This distortion is sufficiently large to produce two equivalent semiconductive *D*_2h_ minima connected through a low-energy *D*_4h_ gapless transition structure, which can be captured with DFT only if a hybrid functional with a sufficient amount of exact exchange is used.

Exploring many first transition row metalloporphenes, we find a large variety of diamagnetic and paramagnetic semiconductors and metallic conductors, unraveling an unexplored landscape of tunable, fully conjugated 2D materials that may show interesting electronic, mechanical, and magnetic properties. It is our hope that the classification presented here will serve as both a motivation and a guiding beacon for further investigation of these systems.

More generally, we have accounted for a large variety of metalloporphene band structures and properties related to them by applying the concepts of antiaromaticity and (Baird) aromaticity. Antiaromatic motifs in materials are desirable as they are often associated with low redox potentials,^60^ open-shell character,^61^ and high carrier mobility,^62^ and this work is a step toward a deeper understanding of their electronic structure.

## Methods

### DFT Calculation Details

All calculations were done with the VASP code.^63−65^ Geometry optimizations were done using the PBE38 functional with D3BJ dispersion corrections, with a planewave kinetic energy cutoff of 420 eV and a 6 × 6 × 1 gamma-centered grid of reciprocal space points. The polymer was positioned in the *xy* plane, and 10 Å of vacuum was added in *z* (13 Å when axial ligands were present). Total energies were converged to 0.1 meV. Finally, a single-point calculation was done at an 8 × 8 × 1 *k* grid to evaluate the electronic structure and total energy more accurately; the output of this calculation was used in subsequent analysis.

The vacuum level was determined using Vaspkit.^66^ Band diagrams, effective masses, and electron mobilities were obtained by interpolating band energies obtained at the 8 × 8 × 1 *k* grid using BoltzTrap2^67^; to obtain an estimate of conductivity, the constant relaxation time approximation (τ = 10^–14^ s) was employed.

The HOMA (harmonic oscillator model of aromaticity) index was calculated according to
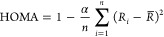
where α = 1682.792, *R*_*i*_ is the length of the *i*th C–C bond, and *R̅* is the average C–C bond length in ^**1**^**TiP** (in Å). α is chosen so that the HOMA value of the *D*_2h_ geometry of ^**1**^**MgP**, which has the highest amount of bond length variation, is equal to 0.

## Data Availability

All optimized geometries (POSCAR) and parsable output files (vasprun.xml) are available as a Zenodo open access data set (doi: 10.5281/zenodo.7580003). Figures of all computed band diagrams and density of state plots are available in the Supporting Information and at https://metalloporphene-electronic-structure.onrender.com/

## Supporting Information Available

Computed band diagrams, density of state plots, and related properties of all metalloporphenes (PDF)

## References

[ref1] Wang W.; Zhao W.; Xu H.; Liu S.; Huang W.; Zhao Q. Fabrication of ultra-thin 2d covalent organic framework nanosheets and their application in functional electronic devices. Coord. Chem. Rev. 2021, 429, 213616 10.1016/j.ccr.2020.213616.

[ref2] Dong R.; Zhang T.; Feng X. Interface-assisted synthesis of 2d materials: Trend and challenges. Chem. Rev. 2018, 118, 6189–6235. 10.1021/acs.chemrev.8b00056. 29912554

[ref3] Colson J. W.; Dichtel W. R. Rationally synthesized two-dimensional polymers. Nat. Chem. 2013, 5, 453–465. 10.1038/nchem.1628. 23695626

[ref4] Payamyar P.; King B. T.; Öttinger H. C.; Schlüter A. D. Two-dimensional polymers: Concepts and perspectives. Chem. Commun. 2016, 52, 18–34. 10.1039/C5CC07381B. 26522010

[ref5] Cai Z.; Liu B.; Zou X.; Cheng H.-M. Chemical vapor deposition growth and applications of two-dimensional materials and their heterostructures. Chem. Rev. 2018, 118, 6091–6133. 10.1021/acs.chemrev.7b00536. 29384374

[ref6] Zeng M.; Xiao Y.; Liu J.; Yang K.; Fu L. Exploring two-dimensional materials toward the next-generation circuits: From monomer design to assembly control. Chem. Rev. 2018, 118, 6236–6296. 10.1021/acs.chemrev.7b00633. 29381058

[ref7] Jin H.; Guo C.; Liu X.; Liu J.; Vasileff A.; Jiao Y.; Zheng Y.; Qiao S.-Z. Emerging two-dimensional nanomaterials for electrocatalysis. Chem. Rev. 2018, 118, 6337–6408. 10.1021/acs.chemrev.7b00689. 29552883

[ref8] Ares P.; Novoselov K. S. Recent advances in graphene and other 2d materials. Nano Materials Science 2022, 4, 3–9. 10.1016/j.nanoms.2021.05.002.

[ref9] Novoselov K. S.; Fal′ko V. I.; Colombo L.; Gellert P. R.; Schwab M. G.; Kim K. A roadmap for graphene. Nature 2012, 490, 192–200. 10.1038/nature11458. 23060189

[ref10] Allen M. J.; Tung V. C.; Kaner R. B. Honeycomb carbon: A review of graphene. Chem. Rev. 2010, 110, 132–145. 10.1021/cr900070d. 19610631

[ref11] Pumera M.; Sofer Z. Towards stoichiometric analogues of graphene: Graphane, fluorographene, graphol, graphene acid and others. Chem. Soc. Rev. 2017, 46, 4450–4463. 10.1039/C7CS00215G. 28524920

[ref12] Matochová D.; Medved’ M.; Bakandritsos A.; Steklý T.; Zbořil R.; Otyepka M. 2d chemistry: Chemical control of graphene derivatization. J. Phys. Chem. Lett. 2018, 9, 3580–3585. 10.1021/acs.jpclett.8b01596. 29890828 PMC6038093

[ref13] Magnera T. F.; Dron P. I.; Bozzone J. P.; Jovanovic M.; Rončević I.; Tortorici E.; Bu W.; Miller E. M.; Rogers C. T.; Michl J. Porphene and porphite as porphyrin analogs of graphene and graphite. Nat. Commun. 2023, 14, 6308 10.1038/s41467-023-41461-w. 37813887 PMC10562370

[ref14] Ono Y.; Hamano T. Peierls distortion in two-dimensional tight-binding model. J. Phys. Soc. Jpn. 2000, 69, 1769–1776. 10.1143/JPSJ.69.1769.

[ref15] Kertesz M.; Choi C. H.; Yang S. Conjugated polymers and aromaticity. Chem. Rev. 2005, 105, 3448–3481. 10.1021/cr990357p. 16218558

[ref16] Popov I. A.; Bozhenko K. V.; Boldyrev A. I. Is graphene aromatic?. Nano Research 2012, 5, 117–123. 10.1007/s12274-011-0192-z.

[ref17] Zdetsis A. D.; Economou E. N. A pedestrian approach to the aromaticity of graphene and nanographene: Significance of huckel’s (4n+2)π electron rule. J. Phys. Chem. C 2015, 119, 16991–17003. 10.1021/acs.jpcc.5b04311.

[ref18] Nakamura Y.; Aratani N.; Shinokubo H.; Takagi A.; Kawai T.; Matsumoto T.; Yoon Z. S.; Kim D. Y.; Ahn T. K.; Kim D.; et al. A directly fused tetrameric porphyrin sheet and its anomalous electronic properties that arise from the planar cyclooctatetraene core. J. Am. Chem. Soc. 2006, 128, 4119–4127. 10.1021/ja057812l. 16551121

[ref19] Yamaguchi Y. Theoretical study of two-dimensionally fused zinc porphyrins: Dft calculations. Int. J. Quantum Chem. 2009, 109, 1584–1597. 10.1002/qua.21998.

[ref20] Yamaguchi Y. Transport properties of two-dimensionally fused zinc porphyrins from linear-response approach. Int. J. Quantum Chem. 2011, 111, 3230–3238. 10.1002/qua.22717.

[ref21] Yamaguchi Y. Theoretical prediction of electronic structures of fully pi-conjugated zinc oligoporphyrins with curved surface structures. J. Chem. Phys. 2004, 120, 7963–7970. 10.1063/1.1690759. 15267712

[ref22] Posligua V.; Aziz A.; Haver R.; Peeks M. D.; Anderson H. L.; Grau-Crespo R. Band structures of periodic porphyrin nanostructures. J. Phys. Chem. C 2018, 122, 23790–23798. 10.1021/acs.jpcc.8b08131.

[ref23] Tan J.; Li W.; He X.; Zhao M. Stable ferromagnetism and half-metallicity in two-dimensional polyporphyrin frameworks. RSC Adv. 2013, 3, 7016–7022. 10.1039/c3ra40502h.

[ref24] Liu J.-H.; Yang L.-M.; Ganz E. Electrocatalytic reduction of co2 by two-dimensional transition metal porphyrin sheets. Journal of Materials Chemistry A 2019, 7, 11944–11952. 10.1039/C9TA01188A.

[ref25] Glöcklhofer F. Concealed antiaromaticity. ChemRxiv 2023, 10.26434/chemrxiv-2023-hnl0w-v2.

[ref26] Ono S. Two-dimensional square lattice polonium stabilized by the spin–orbit coupling. Sci. Rep. 2020, 10, 11810 10.1038/s41598-020-68877-4. 32678271 PMC7366658

[ref27] Nevalaita J.; Koskinen P. Atlas for the properties of elemental two-dimensional metals. Phys. Rev. B 2018, 97, 035411 10.1103/PhysRevB.97.035411.

[ref28] Arovas D. P.; Berg E.; Kivelson S. A.; Raghu S. The hubbard model. Annual Review of Condensed Matter Physics 2022, 13, 239–274. 10.1146/annurev-conmatphys-031620-102024.

[ref29] Zaffran J.; Caspary Toroker M. Benchmarking density functional theory based methods to model niooh material properties: Hubbard and van der waals corrections vs hybrid functionals. J. Chem. Theory Comput. 2016, 12, 3807–3812. 10.1021/acs.jctc.6b00657. 27420033

[ref30] Cohen A. J.; Mori-Sánchez P.; Yang W. Insights into current limitations of density functional theory. Science 2008, 321, 792–794. 10.1126/science.1158722. 18687952

[ref31] Perdew J. P.; Burke K.; Ernzerhof M. Generalized gradient approximation made simple. Phys. Rev. Lett. 1996, 77, 3865–3868. 10.1103/PhysRevLett.77.3865. 10062328

[ref32] Baryshnikov G. V.; Valiev R. R.; Kuklin A. V.; Sundholm D.; Ågren H. Cyclo[18]carbon: Insight into electronic structure, aromaticity, and surface coupling. J. Phys. Chem. Lett. 2019, 10, 6701–6705. 10.1021/acs.jpclett.9b02815. 31609631

[ref33] Deng J.-R.; Bradley D.; Jirásek M.; Anderson H. L.; Peeks M. D. Correspondence on “how aromatic are molecular nanorings? The case of a six-porphyrin nanoring”**. Angew. Chem., Int. Ed. 2022, 61, e202201231 10.1002/anie.202201231. 35818688

[ref34] Karas L. J.; Jalife S.; Viesser R. V.; Soares J. V.; Haley M. M.; Wu J. I. Tetra-tert-butyl-s-indacene is a bond-localized c2h structure and a challenge for computational chemistry. Angew. Chem., Int. Ed. 2023, 62, e202307379 10.1002/anie.202307379. PMC10528983 37467313

[ref35] Parthey M.; Kaupp M. Quantum-chemical insights into mixed-valence systems: Within and beyond the robin–day scheme. Chem. Soc. Rev. 2014, 43, 5067–5088. 10.1039/C3CS60481K. 24781049

[ref36] Renz M.; Theilacker K.; Lambert C.; Kaupp M. A reliable quantum-chemical protocol for the characterization of organic mixed-valence compounds. J. Am. Chem. Soc. 2009, 131, 16292–16302. 10.1021/ja9070859. 19831383

[ref37] Kröncke S.; Herrmann C. Toward a first-principles evaluation of transport mechanisms in molecular wires. J. Chem. Theory Comput. 2020, 16, 6267–6279. 10.1021/acs.jctc.0c00667. 32886502

[ref38] Herrmann C. Electronic communication as a transferable property of molecular bridges?. J. Phys. Chem. A 2019, 123, 10205–10223. 10.1021/acs.jpca.9b05618. 31380640

[ref39] Tanaka Y.; Takahashi H.; Akita M. Estimation of electron distribution over dinuclear organometallic molecular wires by “ir tag” analysis of ancillary acyl-cp ligands. ACS Organic & Inorganic Au 2022, 2, 327–342. 10.1021/acsorginorgau.2c00005. 36855590 PMC9955175

[ref40] Grimme S.; Hansen A.; Brandenburg J. G.; Bannwarth C. Dispersion-corrected mean-field electronic structure methods. Chem. Rev. 2016, 116, 5105–5154. 10.1021/acs.chemrev.5b00533. 27077966

[ref41] Santra G.; Martin J. M. L. What types of chemical problems benefit from density-corrected dft? A probe using an extensive and chemically diverse test suite. J. Chem. Theory Comput. 2021, 17, 1368–1379. 10.1021/acs.jctc.0c01055. 33625863 PMC8028055

[ref42] Monino E.; Boggio-Pasqua M.; Scemama A.; Jacquemin D.; Loos P.-F. Reference energies for cyclobutadiene: Automerization and excited states. J. Phys. Chem. A 2022, 126, 4664–4679. 10.1021/acs.jpca.2c02480. 35820169

[ref43] Wenthold P. G.; Hrovat D. A.; Borden W. T.; Lineberger W. C. Transition-state spectroscopy of cyclooctatetraene. Science 1996, 272, 1456–1459. 10.1126/science.272.5267.1456. 8662467

[ref44] Krygowski T. M.; Szatylowicz H.; Stasyuk O. A.; Dominikowska J.; Palusiak M. Aromaticity from the viewpoint of molecular geometry: Application to planar systems. Chem. Rev. 2014, 114, 6383–6422. 10.1021/cr400252h. 24779633

[ref45] Kruszewski J.; Krygowski T. M. Definition of aromaticity basing on the harmonic oscillator model. Tetrahedron Lett. 1972, 13, 3839–3842. 10.1016/S0040-4039(01)94175-9.

[ref46] Zhou Z.; Petrukhina M. A. Planar, curved and twisted molecular nanographenes: Reduction-induced alkali metal coordination. Coord. Chem. Rev. 2023, 486, 215144 10.1016/j.ccr.2023.215144.

[ref47] Zabula A. V.; Spisak S. N.; Filatov A. S.; Rogachev A. Y.; Petrukhina M. A. Record alkali metal intercalation by highly charged corannulene. Acc. Chem. Res. 2018, 51, 1541–1549. 10.1021/acs.accounts.8b00141. 29874040

[ref48] Zhu H.; Chen Q.; Rončević I.; Christensen K. E.; Anderson H. L. Anthracene-porphyrin nanoribbons. Angew. Chem., Int. Ed. 2023, 31, e202307035 10.1002/anie.202307035. 37293835

[ref49] Barker J. E.; Dressler J. J.; Cárdenas Valdivia A.; Kishi R.; Strand E. T.; Zakharov L. N.; MacMillan S. N.; Gómez-García C. J.; Nakano M.; Casado J.; Haley M. M. Molecule isomerism modulates the diradical properties of stable singlet diradicaloids. J. Am. Chem. Soc. 2020, 142, 1548–1555. 10.1021/jacs.9b11898. 31876145

[ref50] Mishra S.; Vilas-Varela M.; Lieske L.-A.; Ortiz R.; Fatayer S.; Rončević I.; Albrecht F.; Frederiksen T.; Peña D.; Gross L. Bistability between pi-diradical open-shell and closed-shell states in indeno[1,2-a]fluorene. arXiv 2023. 10.48550/arXiv.2303.04483. PMC11087267 38332330

[ref51] Tachikawa H. Jahn–teller effect of the benzene radical cation: A direct ab initio molecular dynamics study. J. Phys. Chem. A 2018, 122, 4121–4129. 10.1021/acs.jpca.8b00292. 29641198

[ref52] Bazante A. P.; Davidson E. R.; Bartlett R. J. The benzene radical anion: A computationally demanding prototype for aromatic anions. J. Chem. Phys. 2015, 142, 204304 10.1063/1.4921261. 26026444

[ref53] García de Arquer F. P.; Talapin D. V.; Klimov V. I.; Arakawa Y.; Bayer M.; Sargent E. H. Semiconductor quantum dots: Technological progress and future challenges. Science 2021, 373, eaaz8541 10.1126/science.aaz8541. 34353926

[ref54] Gaita-Ariño A.; Luis F.; Hill S.; Coronado E. Molecular spins for quantum computation. Nat. Chem. 2019, 11, 301–309. 10.1038/s41557-019-0232-y. 30903036

[ref55] Hirohata A.; Yamada K.; Nakatani Y.; Prejbeanu I.-L.; Diény B.; Pirro P.; Hillebrands B. Review on spintronics: Principles and device applications. J. Magn. Magn. Mater. 2020, 509, 166711 10.1016/j.jmmm.2020.166711.

[ref56] Zatko V.; Dubois S. M. M.; Godel F.; Galbiati M.; Peiro J.; Sander A.; Carretero C.; Vecchiola A.; Collin S.; Bouzehouane K.; et al. Almost perfect spin filtering in graphene-based magnetic tunnel junctions. ACS Nano 2022, 16, 14007–14016. 10.1021/acsnano.2c03625. 36068013 PMC9527810

[ref57] Lakatos I. The methodology of scientific research programmes: Philosophical papers; Cambridge University Press, 1978. 10.1017/CBO9780511621123.

[ref58] Zhou C.; Gagliardi L.; Truhlar D. G. Multiconfiguration pair-density functional theory for iron porphyrin with cas, ras, and dmrg active spaces. J. Phys. Chem. A 2019, 123, 3389–3394. 10.1021/acs.jpca.8b12479. 30763100

[ref59] Plutnar J.; Bednarova L.; Cisarova I.; Dracinsky M.; Krepsova L.; Tarabek J.; Michl J. Parent porphyrin (porphine) and its complexes with 3d metals. ChemRxiv 2023, 10.26434/chemrxiv-2023-tf6b4.

[ref60] Lin Z.; Lin L.; Zhu J.; Wu W.; Yang X.; Sun X. An anti-aromatic covalent organic framework cathode with dual-redox centers for rechargeable aqueous zinc batteries. ACS Appl. Mater. Interfaces 2022, 14, 38689–38695. 10.1021/acsami.2c08170. 35975747

[ref61] Di Giovannantonio M.; Eimre K.; Yakutovich A. V.; Chen Q.; Mishra S.; Urgel J. I.; Pignedoli C. A.; Ruffieux P.; Müllen K.; Narita A.; Fasel R. On-surface synthesis of antiaromatic and open-shell indeno[2,1-b]fluorene polymers and their lateral fusion into porous ribbons. J. Am. Chem. Soc. 2019, 141, 12346–12354. 10.1021/jacs.9b05335. 31309832

[ref62] Ukai S.; Takamatsu A.; Nobuoka M.; Tsutsui Y.; Fukui N.; Ogi S.; Seki S.; Yamaguchi S.; Shinokubo H. A supramolecular polymer constituted of antiaromatic niii norcorroles. Angew. Chem., Int. Ed. 2022, 61, e202114230 10.1002/anie.202114230. 34862699

[ref63] Kresse G.; Furthmüller J. Efficiency of ab-initio total energy calculations for metals and semiconductors using a plane-wave basis set. Comput. Mater. Sci. 1996, 6, 15–50. 10.1016/0927-0256(96)00008-0. 9984901

[ref64] Kresse G.; Furthmüller J. Efficient iterative schemes for ab initio total-energy calculations using a plane-wave basis set. Phys. Rev. B 1996, 54, 11169–11186. 10.1103/PhysRevB.54.11169. 9984901

[ref65] Kresse G.; Hafner J. Ab initio molecular dynamics for liquid metals. Phys. Rev. B 1993, 47, 558–561. 10.1103/PhysRevB.47.558. 10004490

[ref66] Wang V.; Xu N.; Liu J.-C.; Tang G.; Geng W.-T. Vaspkit: A user-friendly interface facilitating high-throughput computing and analysis using vasp code. Comput. Phys. Commun. 2021, 267, 108033 10.1016/j.cpc.2021.108033.

[ref67] Madsen G. K. H.; Carrete J.; Verstraete M. J. Boltztrap2, a program for interpolating band structures and calculating semi-classical transport coefficients. Comput. Phys. Commun. 2018, 231, 140–145. 10.1016/j.cpc.2018.05.010.

