# Supplementary material for: Electronic Structure of Metalloporphenes, Antiaromatic Analogues of Graphene
Source: J Am Chem Soc. 2024 Jan 31;146(6):3992–4000. doi: 10.1021/jacs.3c12079 (PMC10870706; doi:10.1021/jacs.3c12079)
Supplement: Supplementary file 1 — ja3c12079_si_001.pdf [file ja3c12079_si_001.pdf]

Supporting Information for Electronic Structure of Metalloporphenes, Antiaromatic Analogs of Graphene

Ivan Pavlak,[a] Lujo Matasović,[b] Eric A. Buchanan,[c] Josef Michl,[c,d] and Igor Rončević*[d,e]

[a] Department of Chemistry, Faculty of Science, University of Zagreb, Horvatovac 102A, 10000 Zagreb, Croatia

[b] Cavendish Laboratory, Department of Physics, University of Cambridge, J. J. Thomson Avenue, Cambridge, CB3 0HE, UK

[c] Department of Chemistry and Biochemistry, University of Colorado, 80309-0215 Boulder, CO, United States

[d] Institute of Organic Chemistry and Biochemistry of the CAS, Flemingovo nám. 2, 16610 Prague 6, Czech Republic

[e] Department of Chemistry, University of Oxford, Chemistry Research Laboratory, Oxford OX1 3TA, UK

Table of Contents

TABLE S1 2

FUNCTIONAL CHOICE 3

DEFECTS 5

FROM PORPHYRIN TO PORPHENE 7

TABLE S3 9

TABLE S4 10

INCAR FILES 11

PRELIMINARY OPTIMIZATION 11
PRODUCTION-LEVEL OPTIMIZATION 12
PRELIMINARY ELECTRONIC STRUCTURE 13
PRODUCTION-LEVEL ELECTRONIC STRUCTURE 14

BAND STRUCTURES OF ALL METALLOPORPHENES 15

GROUP −1 15
GROUP 0^{TT} 18
GROUP 0^{T} 22
GROUP 0^{U} 28
GROUP 0^{UU} 30
GROUP 1 32
GROUP 2 34

Table S1. Sensitivity of metalloporphene properties to the amount of exact exchange (% EE) included in the DFT functional.

DFT	PBE0 (25% EE)	← PBE38 (37.5% EE) →	PBE50 (50% EE)
group	remain **−1**<br>σ[a] increased by ~3%	**−1**	remain **−1**<br>σ decreased by ~30%
	remain $\mathbf{0}^{tt}$<br>σ increased by ~5%	$\mathbf{0}^{tt}$	remain $\mathbf{0}^{tt}$<br>σ decreased by ~5%
	remain $\mathbf{0}^{t}$<br>σ increased by ~19%	$\mathbf{0}^{t}$	~50% become $\mathbf{0}^{U}$<br>with E_g[b] ≈ 0.6 eV
	~50% become $\mathbf{0}^{t}$ or<br>E_g reduced by ~20%	$\mathbf{0}^{U}$	remain $\mathbf{0}^{U}$<br>E_g increased by ~42%
	remain $\mathbf{0}^{UU}$<br>E_g reduced by ~27%	$\mathbf{0}^{UU}$	remain $\mathbf{0}^{UU}$<br>E_g increased by ~31%
	remain **1**<br>σ decreased by ~49%	**1**	remain **1**<br>σ increased by ~20%
	remain **2**<br>E_g reduced by ~22%	**2**	remain **2**<br>E_g increased by ~24%

[a] conductivity
[b] direct band gap

Functional choice

When choosing the functional to use, we considered the following properties:

(a) good description of symmetry breaking

(b) good account of the extent of electron (de)localization

(c) numerical stability, few adjustable parameters, long-range corrections

(a) Symmetry breaking. In order to accurately capture symmetry breaking in π-conjugated compounds, a functional should describe both σ- and π-bonding with comparable accuracy.[1] As many contemporary functionals are geared towards the description of molecular properties,(e.g. 2) which are largely determined by the orbitals close to the Fermi energy, they are not guaranteed to give a good description of symmetry breaking. We looked at the % EE at which symmetry breaking occurs in archetypical cases such as cyclo[18]carbon[3] (**1**), tetra-tert-butyl-s-indacene[4] (**2**), and the hexacation of the butadiyne-linked six-porphyrin nanoring[5] (**3**). Our results (Table S2) show that at 37.5% EE, symmetry breaking occurs in all three cases, consistent with experimental results and previous studies. Naturally, symmetry breaking persists at higher (50%) amounts of EE, but using such high amounts of EE usually results in worse performance, which can be attributed to increased errors due to static correlation and worse description of dynamic correlation,[6] although highly tuned empirical functionals can remedy this (e.g. M06-2X, which has 54% exchange).

Table S2. The amount of EE required for symmetry breaking in cyclo[18]carbon (**1**), tetra-tert-butyl-s-indacene (**2**), and the hexacation of the butadiyne-linked six-porphyrin nanoring (**3**). Symmetry-broken point groups are shown in bold.

% EE / compound		0	10	20	25	30	35	37.5	40	50	prev work
point group	**1**	D_{18h}	D_{18h}	D_{18h}	D_{18h}	**D_{9h}**	**D_{9h}**	**D_{9h}**	**D_{9h}**	**D_{9h}**	**D_{9h}**[3]
	**2**	D_{2h}	D_{2h}	D_{2h}	D_{2h}	D_{2h}	**C_{2h}**	**C_{2h}**	**C_{2h}**	**C_{2h}**	**C_{2h}**[4]
	**3**				D_{6h}			**C_{3h}**		**C_{3h}**	**C_{3h}**[5]

Due to the presence of both aromatic and antiaromatic circuits in its structure, we investigated **2** in more detail. In their recent work,[4] Wu and Haley found that an accurate description of chemical shifts in **2** is very challenging for some DFT functionals. Following their protocol (which uses B97D-2 for chemical shift calculations), we find that a very good agreement with experiment (δ_{RMSD} = 0.61 ppm) is obtained if a PBE38 geometry is used, and that using geometries obtained with either more or less EE worsens the agreement of calculated chemical shifts with experiment.

(b) Electron delocalization. When looking at how to describe metalloporphenes, one source of inspiration were mixed-valence compounds. In these systems, the electronic coupling between different sites (i.e. the extent of electron/hole delocalization) can range from essentially zero (Robin-Day class I), to intermediate (Robin-Day class II), and very strong (Robin-Day class III).[7] Renz and Kaup have shown that BLYP35 (35% EE) is not only successful at determining the Robin-Day class of various mixed-valence compounds, but it is also capable of accurately predicting their electronic and optical properties.[8-10] The same functional was also successful for describing electron delocalisation in purely organic molecular wires.[11-13]

(c) Stability. Unfortunately, calculations on metalloporphenes using BLYP35 were prone to numerical instabilities, precluding us from using it for large-scale calculations. However, calculations on a 2×2 ZnP fragment using BLYP35 and PBE38 give very similar results (HOMO-LUMO gap values $E_{HL,BLYP35}$ = 1.65 eV; $E_{HL,BLYP35}$ = 1.74 eV , and BLA values at the cyclooctatetraene ring BLA_{BLYP35} = 0.048 Å and BLA_{PBE38} = 0.053 Å), indicating that PBE38 is a reasonable substitute.

Defects

The positions of investigated defects **a**, **b**, and **c** in a 2×2 supercell of **ZnP** are shown in Figure S1. As geometry optimisations of 2×2 supercells of **ZnP** with C_1 symmetry are prohibitively costly, we optimised smaller model systems and then manually built our defect geometries.

For defect **a** (Figure S2a), one Zn atom was replaced with two hydrogens, which were placed on opposing nitrogens with a bond length of 0.99 Å. Such a defect may be formed if the reinsertion of Zn following the polymerization is incomplete, and experimental evidence suggests that the density of such free-base macrocycles is no larger than 1 in 400.[14] The direct band gap of this system was found to be ~0.49 eV, about half of the value in pure ZnP.

Defects **b** and **c** (Figure S2b,c) correspond to the interruption in the π-conjugation. They were built by taking two neighboring meso (**b**) or beta (**c**) carbons out of the porphene plane by roughly ±0.15 Å for **b** (±0.20 Å in the case of **c**) Å and adding hydrogens to them (1.10 Å) in the axial position, which resulted in C-C-H angles of 105° for **b** (106° in the case of **c**). Defect **b** interrupts only the aromatic circuits and results in a strong shrinkage of the band gap (0.12 eV). Defect c affects both aromatic and antiaromatic circuits and has a relatively smaller effect on the band gap (0.54 eV).

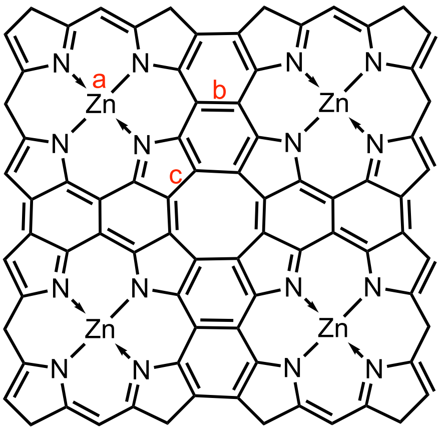

Figure S1. Defects in **ZnP**. (a) corresponds to the replacement of one zinc with two hydrogens, while (b) and (c) describe structures with the highlighted bond hydrogenated.

Figure S2. Band structures of (a) defect **a**, (b) defect **b**, and (c) defect **c** in ZnP.

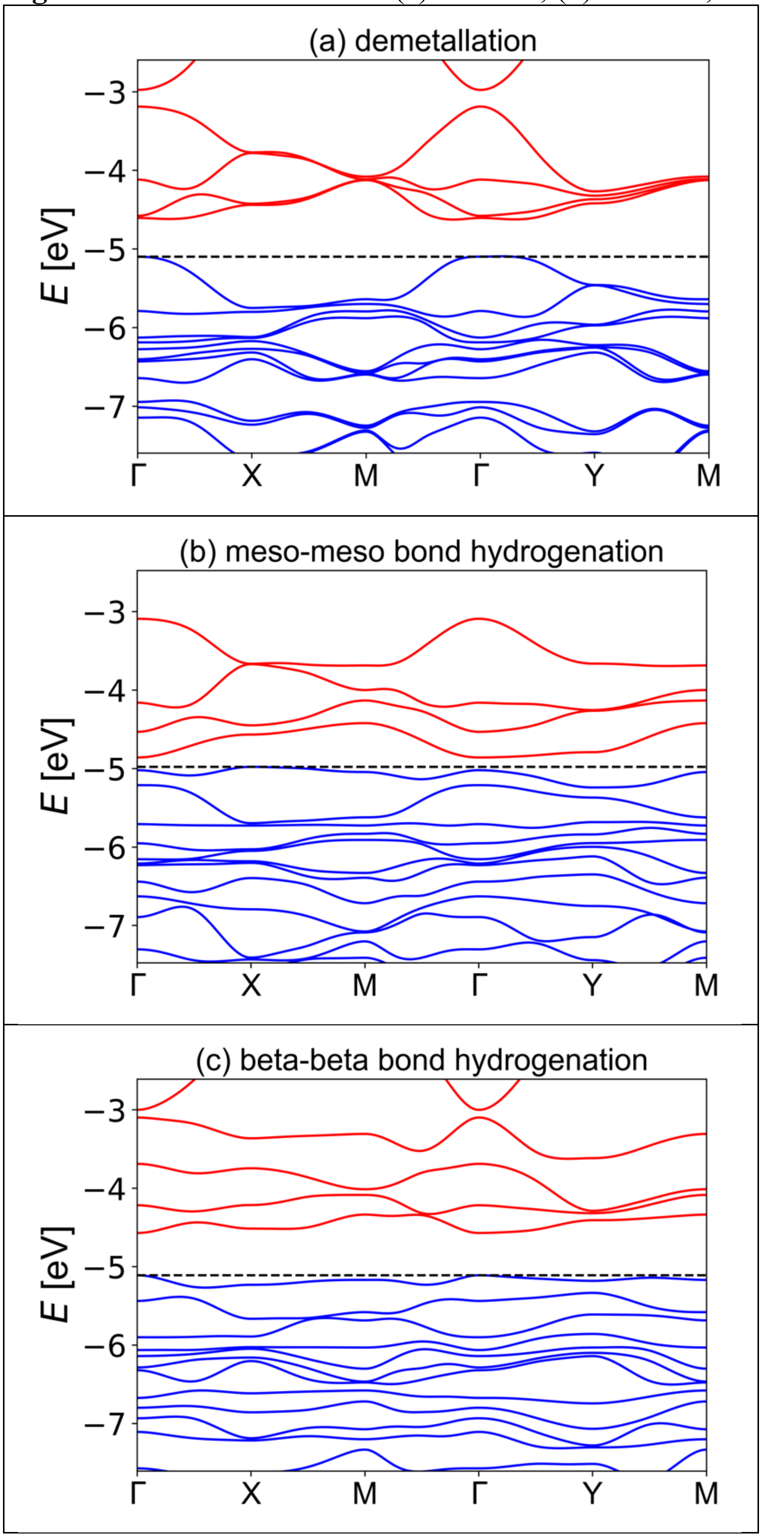

From porphyrin to porphene

To understand how the electronic structure of Zn porphyrin is transformed into Zn porphene, we performed molecular calculations of Zn porphyrin and 2×2 and 3×3 Zn porphene fragments using PBE38 (Figure S3). We note that 3×3 fragment calculation fails when a functional with a low proportion of EE is used (e.g. B3LYP or PBE0), but proceeds without issue if a high-EE functional such as PBE38 or M06-2X (or a range-separated functional such as ωB97X-D) is used. In all cases when the calculation is successful, the geometry optimises to a minimum with alternating bond lengths in the cyclooctatetraene moiety.

Our results show that both the HOMO-LUMO gap and the average amount of bond-length alternation (BLA) in the cyclooctatetraene moieties reach values comparable to those in a polymer for a 3×3 fragment of **ZnP** (Figure S3c,d). Direct comparison is difficult, as a small Gaussian basis was used for molecular calculations, and a larger plane wave-based basis (and pseudopotentials) were used for the polymer.

The electronic structure and antiaromaticity of **ZnP** may be compared to its 2×2 fragment (Figure S3e), where one BLA pattern of orbitals in the cycylooctatetraene moiety is associated with HOMO – 1 and HOMO – 11, and the other to HOMO and LUMO, leading to a paratropic coupling between HOMO – 1 and LUMO and driving the bond-length alternation.

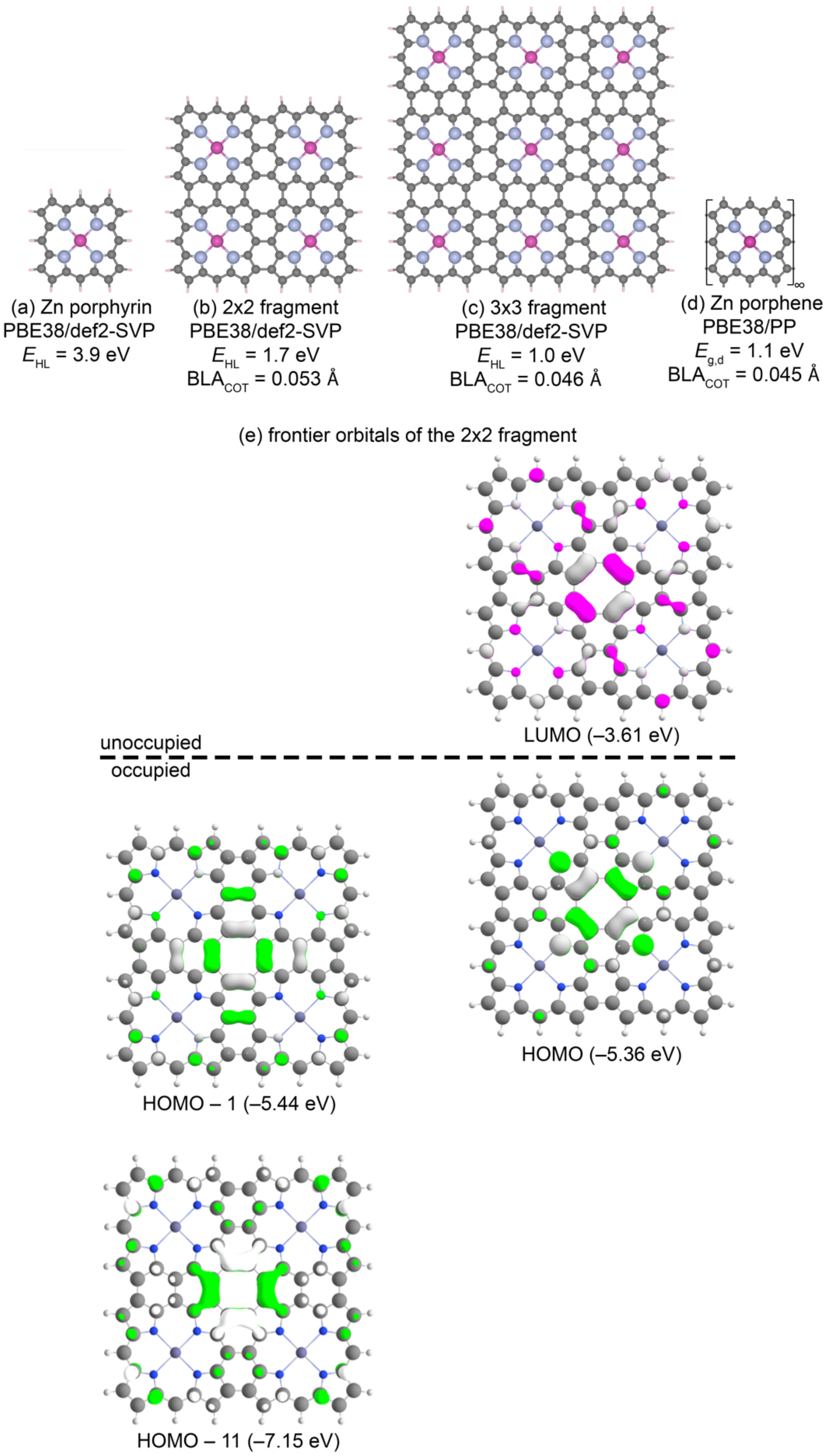

Figure S3. The HOMO-LUMO gap (E_{HL}, or band gap E_g in case of the polymer) and average amount of bond-length alternation in the cyclooctatetraene unit(s) (BLA_{COT}) in (**a**) Zn porphyrin, (**b**) a 2×2 or (**c**) a 2×2 fragment of **ZnP**, and (**d**) in **ZnP**. **(e)** 2×2 fragment orbitals with significant π-density on the cyclooctatetraene unit.

Table S3. Properties of all gapless metalloporphenes.

grp	cmpd	ΔE[a]	W[b]	σ[c]	HOMA
−1	^{2}V(III)$^{+}$Cl$_2$P^{-}	0	5.80	0.33	0.62
	^{5}Fe(III)$^{+}$Cl$_2$P^{-}		6.58	0.83	0.39
	^{4}Co(I)$^{+}$P^{-}		5.53	0.46	0.64
	^{4}NiClP		6.32	0.84	0.32
	^{2}VOP		6.09	0.87	0.65
	^{3}CrOP		5.99	1.01	0.64
	^{2}CoCl$_2$P		6.42	0.87	0.36
	^{6}CoOP		6.42	0.69	0.37
0′′	^{4}FeClP	0	5.73	1.02	0.52
	^{1}CrOP		5.57	0.98	0.62
	^{6}CoCl$_2$P		6.36	1.16	0.43
	^{1}NiCl$_2$P		6.07	1.44	0.25
	^{5}NiCl$_2$P		7.63	0.35	0.38
	^{4}FeClOP		6.32	1.19	0.26
	^{5}FeOP		5.51	1.07	0.57
	^{1}TiCl$_2$P		6.20	0.94	0.01
	^{2}NiClP		6.56	0.90	0.31
	^{3}FeCl$_2$P		6.79	1.13	0.44
	^{4}CoCl$_2$P		7.00	0.37	0.75
	^{4}CoOP		6.35	0.97	0.30
	^{4}CrClP		5.56	1.02	0.60
0′	^{6}FeClP	0.03	5.57	0.49	0.41
	^{1}NiP	0.01	5.08	0.91	0.40
	^{1}CoClP	0.49	5.62	0.98	0.38
	^{2}MnP	1.25	4.02	0.23	0.66
	^{2}CoP	0.21	4.86	0.47	0.40
	^{5}CoClP	0.12	6.26	0.84	0.29
	^{1}VClP	0.05	**5.56**	0.96	0.55
	^{3}VClP	0.06	5.64	0.46	0.46
	^{1}TiOP	0.02	5.80	0.28	0.39
	^{1}FeCl$_2$P	0.05	6.90	0.97	-0.07
	^{1}FeOP	0.83	5.73	1.05	0.46
	^{3}NiCl$_2$P	0.02	**6.26**	1.27	0.42
	^{2}FeClOP	0.01	5.68	1.01	0.58
	^{3}FeO$_2$P	1.54	6.35	1.27	0.45
	^{5}CrP	0.36	4.89	0.55	0.51
	^{2}CuP	0.05	4.90	0.47	0.39
1	^{2}Sc(III)$^{3+}$P^{3-}	0.01	4.65	0.31	0.70
	^{3}Ti(III)$^{3+}$P^{3-}	0.40	4.84	0.26	0.90
	^{4}V(III)$^{3+}$P^{3-}	0.68	4.77	0.23	0.92
	^{6}Mn(III)$^{3+}$P^{3-}	0.14	4.62	0.17	0.90
	^{3}Fe(III)$^{3+}$P^{3-}	0.05	5.29	0.44	0.80
	^{4}Mn(III)$^{3+}$P^{3-}	<0.01	4.93	0.29	0.89
	^{2}Ti(III)$^{3+}$P^{3-}	<0.01	5.10	0.25	0.93

[a] Energy difference between the D_{4h} and D_{2h} minima (eV).[b] work function (eV); [c] conductivity (MS/m)

Table S4. Properties of all semiconductive metalloporphenes.

grp	cmpd.	$E_{g,i}$[a] $E_{g,d}$	ΔE[b]	W[c]	m_{VB}[d] m_{CB}	HOMA
**0**^{U}	**[1]ScClP**	0.31 1.21	0.06	5.77	0.10 0.37	0.02
	**[5]FeP**	0.48 3.07	0.43	4.89	0.17 0.23	0.09
	**[1]MgP**	0.22 1.75	0.1	4.86	0.10 0.33	0.04
	**[1]CrP**	0.41 2.35	3.27	5.26	0.12 0.05	0.28
	**[1]FeP**	0.39 2.01	1.49	5.02	0.10 0.08	0.12
	**[1]FeO₂P**	- 1.15	0.05	5.86	0.17 0.11	0.07
	**[1]ZnP**	0.22 1.13	0.10	4.91	0.18 0.41	0.04
**0**^{UU}	**[2]CrClP**	0.77 2.22	2.78	5.88	0.24 0.16	0.64
	**[3]CrP**	0.61 2.12	0	5.13	0.22 0.15	0.66
	**[3]FeOP**	0.67 1.78	0.16	5.71	0.19 0.15	0.49
	**[2]VP**	0.84 2.1	0.21	5.12	0.38 0.09	0.66
	**[2]FeClP**	1.34 2.28	0	6.06	0.27 0.16	0.56
	**[3]CoClP**	0.82 2.58	0	5.68	0.25 0.20	0.59
**2**	**[1]Ti(VI)$^{4+}$P^{4-}**	2.17 2.28	0	5.54	1.22 0.42	0.74

[a] Indirect ($E_{g,i}$) and direct ($E_{g,d}$) band gap.
[b] Energy difference between the D_{4h} and D_{2h} minima (eV).
[c] work function (eV); [d] valence (m_{VB}) and conduction (m_{CB}) eff. mass

INCAR Files

Preliminary Optimization

```
SYSTEM = Zn2_D2h_0
 PREC = N
 ENCUT = 420
 ALGO = NORMAL
 ISMEAR = 0
 SIGMA = 0.14
 EDIFF = 1E-5
 LREAL = AUTO
 PRECFOCK = FAST
 LHFCALC = .TRUE.
 GGA = PE; AEXX = 0.375
 IVDW = 12
 VDW_S8 = 1.4623
 VDW_A1 = 0.3995
 VDW_A2 = 5.1405

 IBRION = 2
 POTIM = 0.2
 NSW = 100
 EDIFFG = 1E-4
 ISIF = 4

 AMIX     = 0.2
 BMIX     = 0.0001 ! almost zero, but 0 will crash some versions
 AMIX_MAG = 0.8
 BMIX_MAG = 0.0001 ! almost zero, but 0 will crash some versions

 # Magnetism
 ISPIN = 1

 # Other
 LWAVE = .TRUE.
 LCHARG = .FALSE.
```

Production-Level Optimization

```
# General
 SYSTEM = Zn2_D2h_0
 PREC = N
 ENCUT = 420
 ALGO = DAMPED; TIME = 0.15
 ISMEAR = 0
 SIGMA = 0.12
 EDIFF = 1E-5
 LREAL = AUTO
 PRECFOCK = FAST
 LHFCALC = .TRUE.
 GGA = PE; AEXX = 0.375
 IVDW = 12
 VDW_S8 = 1.4623
 VDW_A1 = 0.3995
 VDW_A2 = 5.1405

 IBRION = 2
 POTIM = 0.2
 NSW = 100
 EDIFFG = 1E-4
 ISIF = 4

 # Magnetism
 ISPIN = 1

 # Other
 LWAVE = .TRUE.
 LCHARG = .FALSE.
```

Preliminary Electronic Structure

```
# General
 SYSTEM = Zn2_D2h_0
 PREC = N
 ENCUT = 420
 ALGO = DAMPED; TIME = 0.15
 ISMEAR = -5
 SIGMA = 0.075
 EDIFF = 1E-6
 LREAL = AUTO
 PRECFOCK = FAST
 LHFCALC = .TRUE.
 GGA = PE; AEXX = 0.375
 IVDW = 12
 VDW_S8 = 1.4623
 VDW_A1 = 0.3995
 VDW_A2 = 5.1405
 LOPTICS = .TRUE.; LPEAD = .TRUE.

 # Magnetism
 ISPIN = 1

 # Other
 LWAVE = .TRUE.
 LCHARG = .FALSE.
```

Production-Level Electronic Structure

```
# General
 SYSTEM = Zn2_D2h_0
 PREC = N
 ENCUT  = 420
 ALGO = DAMPED; TIME = 0.15
 ISMEAR = -5
 SIGMA = 0.075
 EDIFF = 1E-6
 LREAL = AUTO
 PRECFOCK = FAST
 LHFCALC = .TRUE.
 GGA = PE; AEXX = 0.375
 IVDW = 12
 VDW_S8 = 1.4623
 VDW_A1 = 0.3995
 VDW_A2 = 5.1405
 LVTOT = .TRUE.
 NBANDS = 200
 LOPTICS = .TRUE.; LPEAD = .TRUE.

 # Magnetism
 ISPIN = 1

 # Other
 LWAVE = .TRUE.
 LCHARG = .FALSE.
```

Band Structures of All Metalloporphenes

Group **–1**

$^{2}V(III)^{+}Cl_2P^{-}$

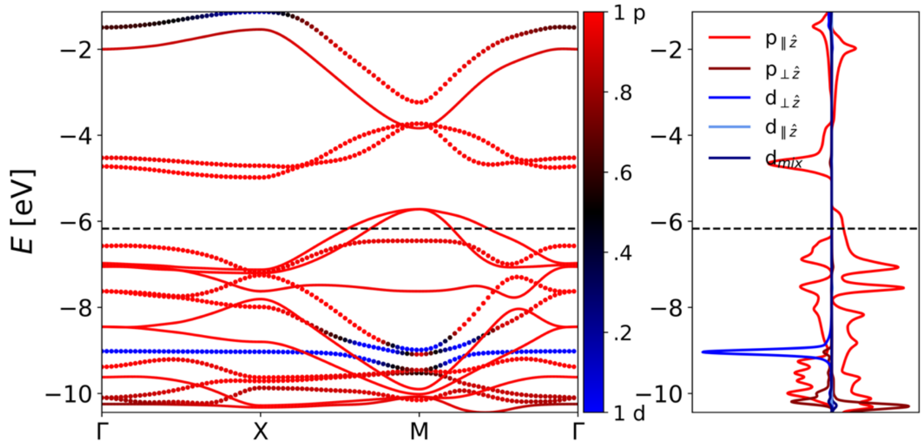

$^{5}Fe(III)^{+}Cl_2P^{-}$

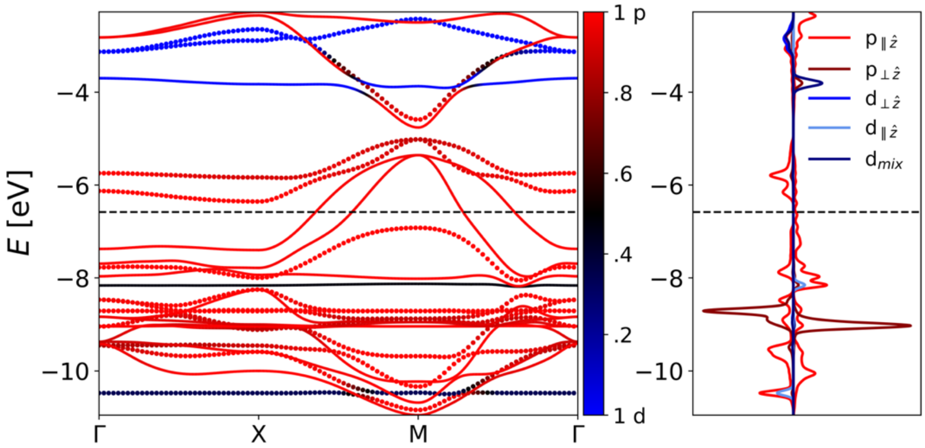

$^{4}Co(I)^{+}P^{-}$

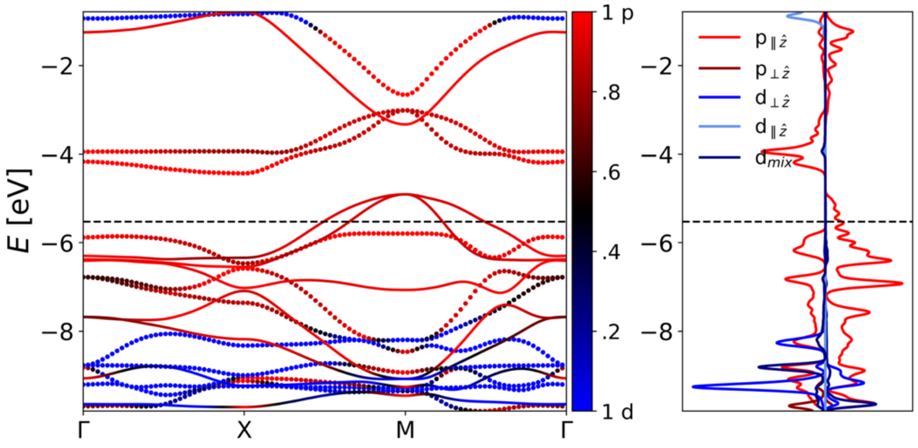

^{4}NiClP

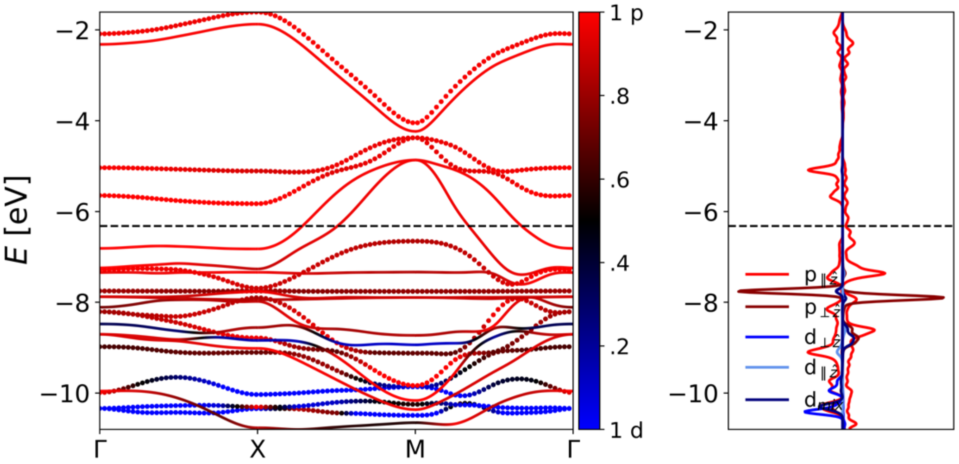

^{2}VOP

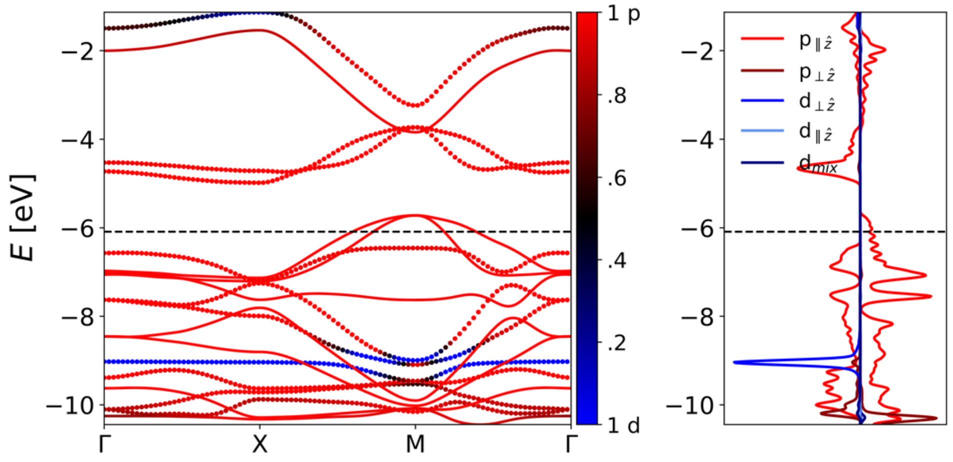

^{3}CrOP

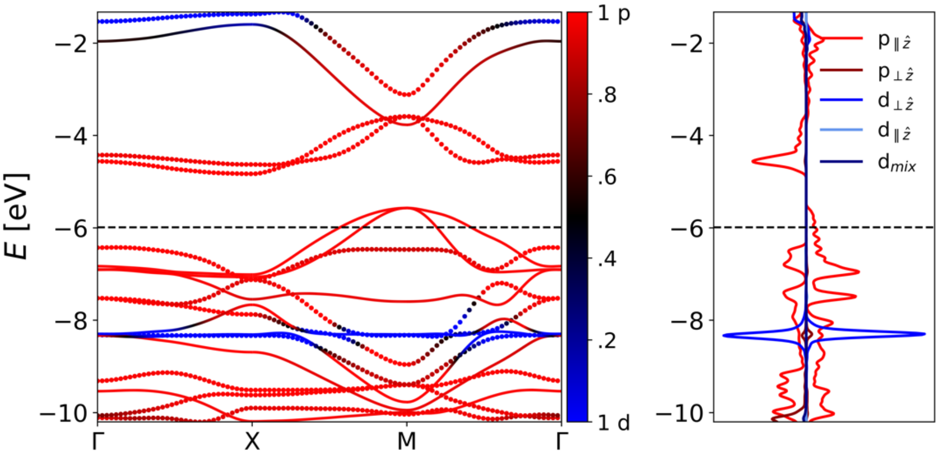

$^{2}\mathrm{CoCl_2P}$

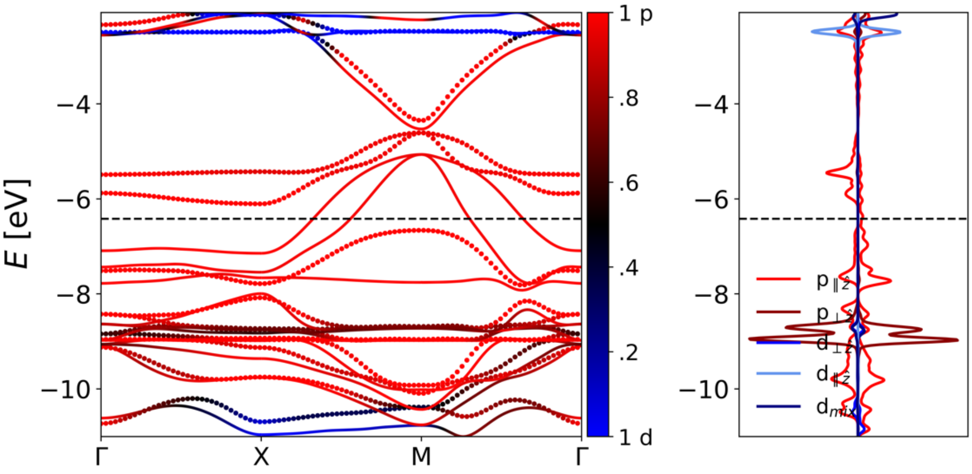

$^{6}\mathrm{CoOP}$

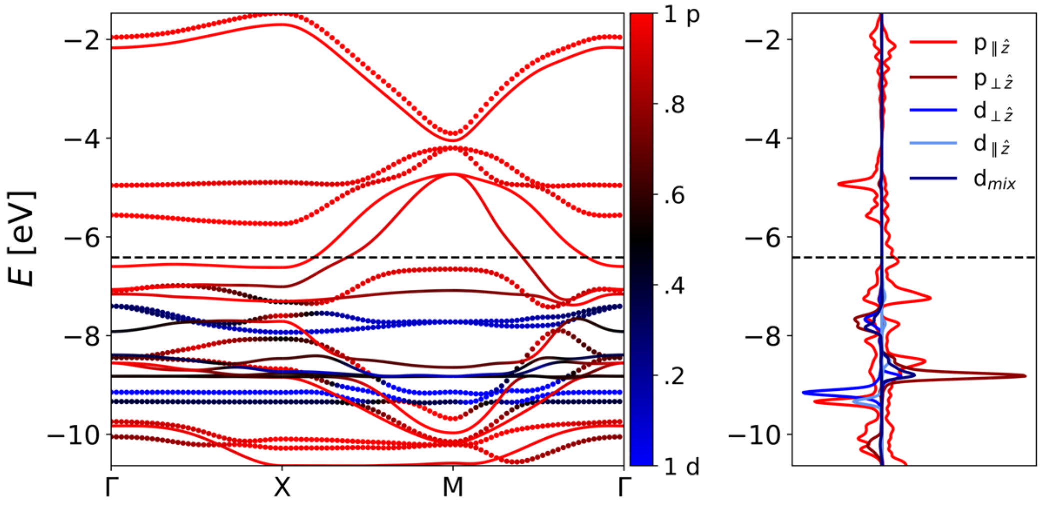

Group **0''**

$^{1}TiCl_2P$

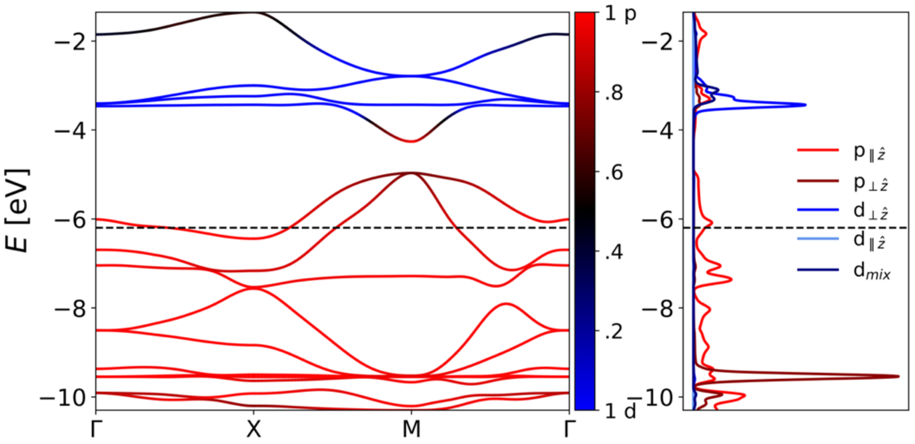

$^{4}CrClP$

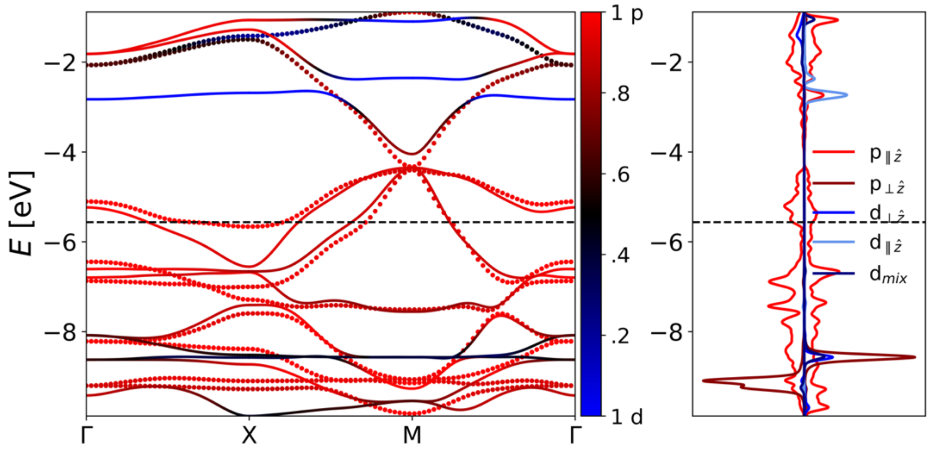

$^{4}FeClP$

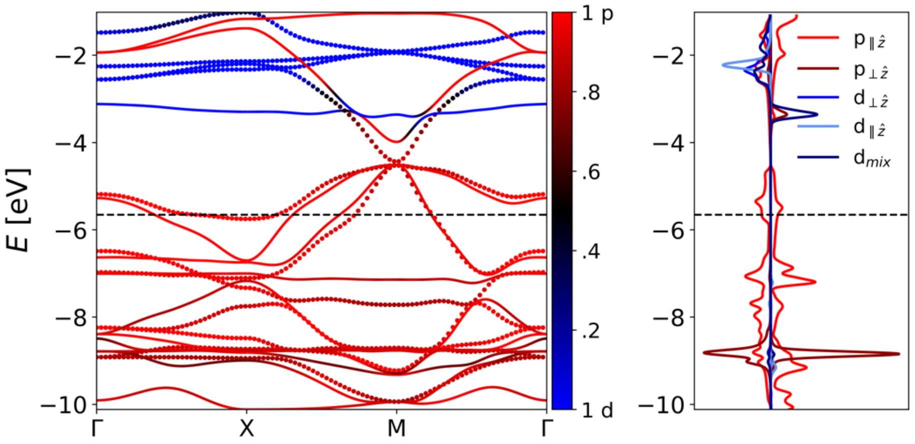

^{1}CrOP

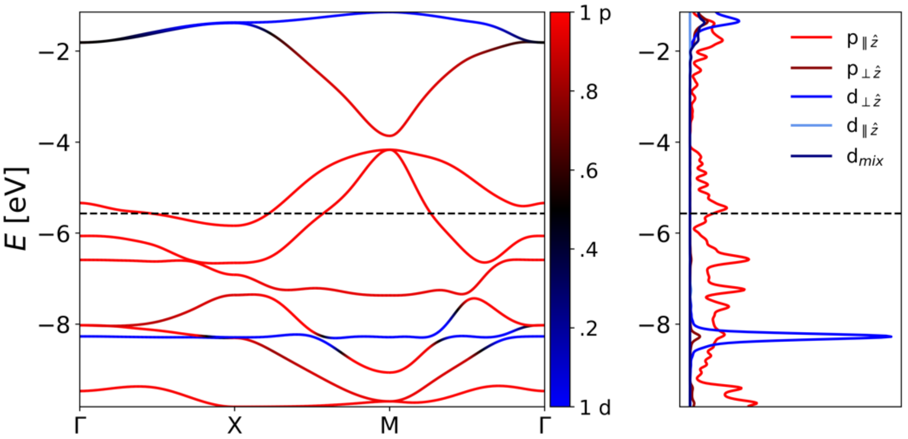

^{6}CoCl$_2$P

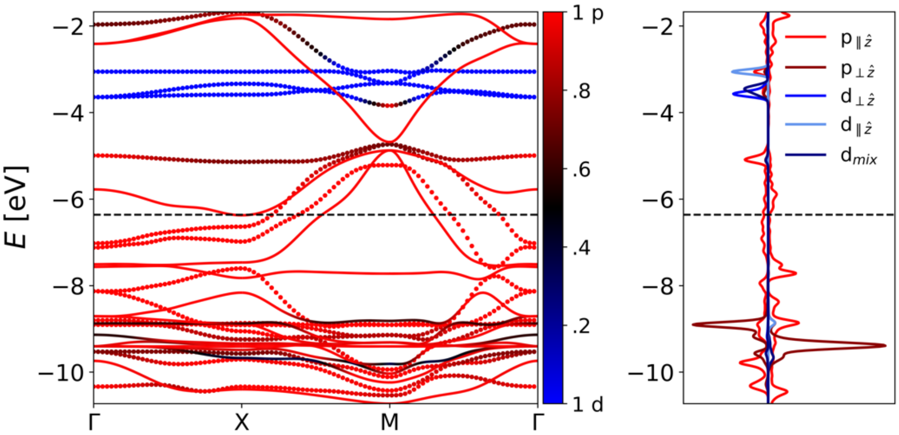

^{1}NiCl$_2$P

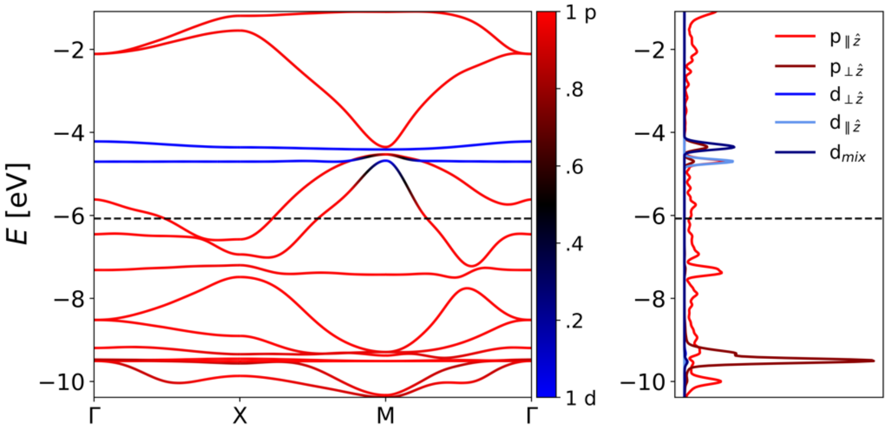

^{5}NiCl$_2$P

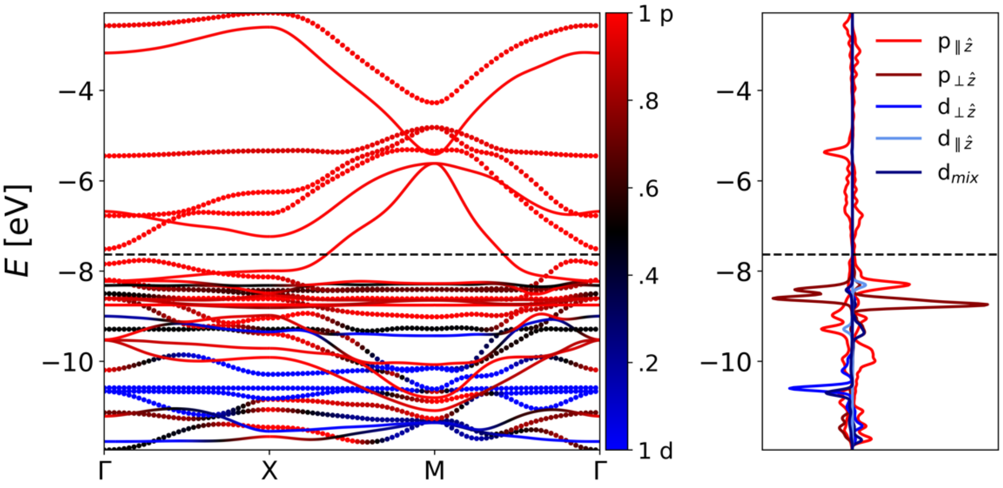

^{4}FeClOP

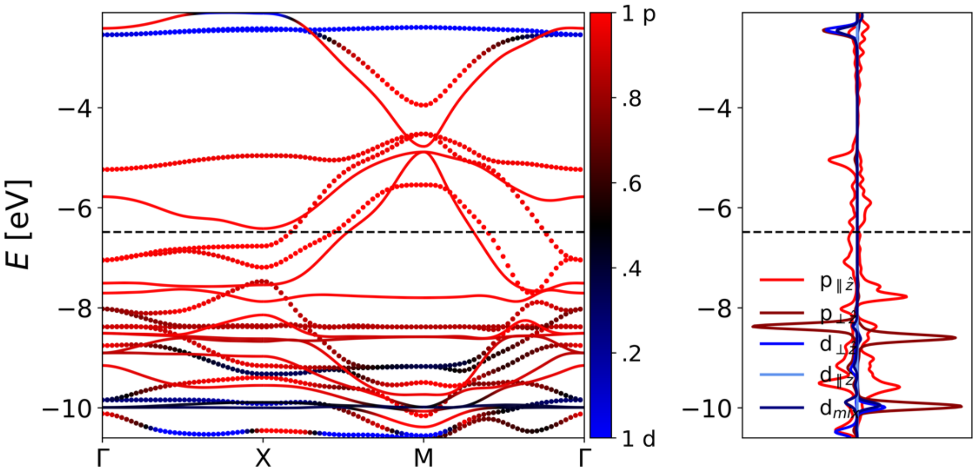

^{2}NiClP

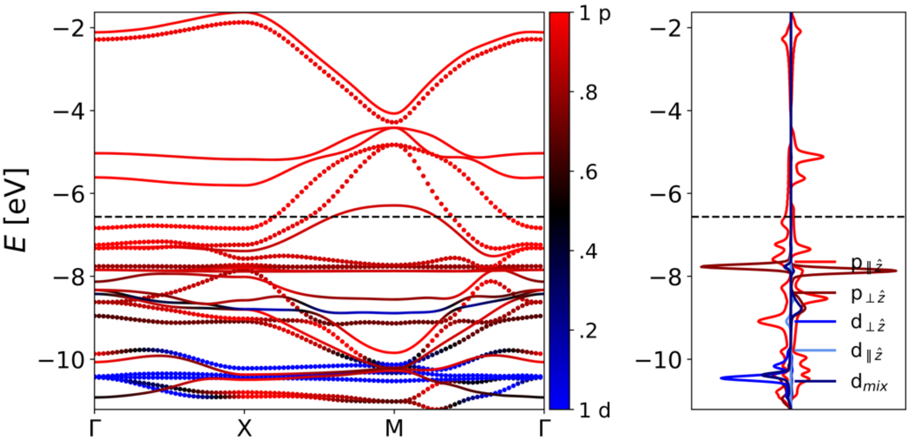

$^{3}FeCl_2P$

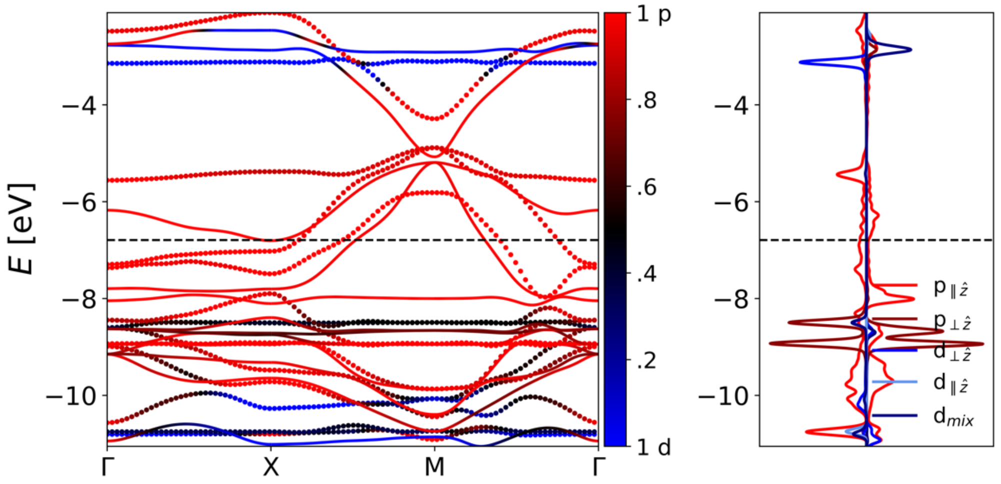

$^{4}CoCl_2P$

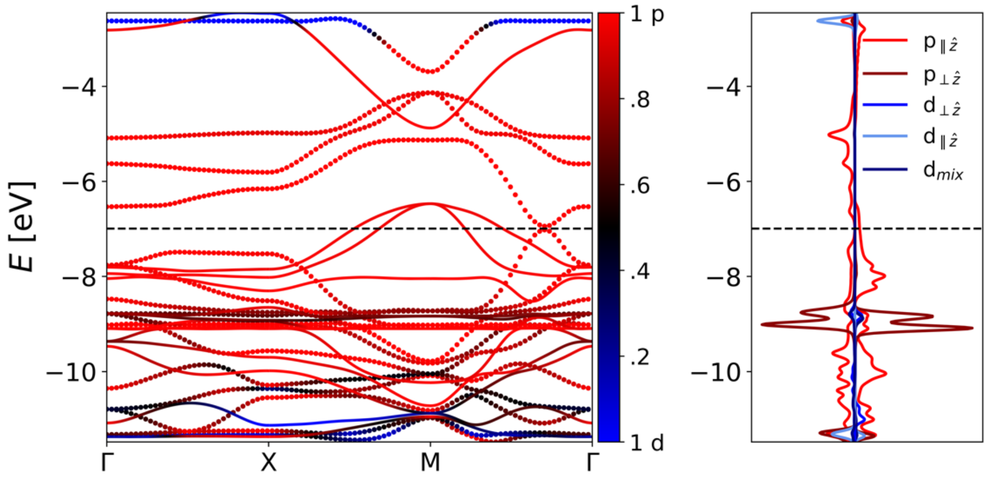

$^{4}CoOP$

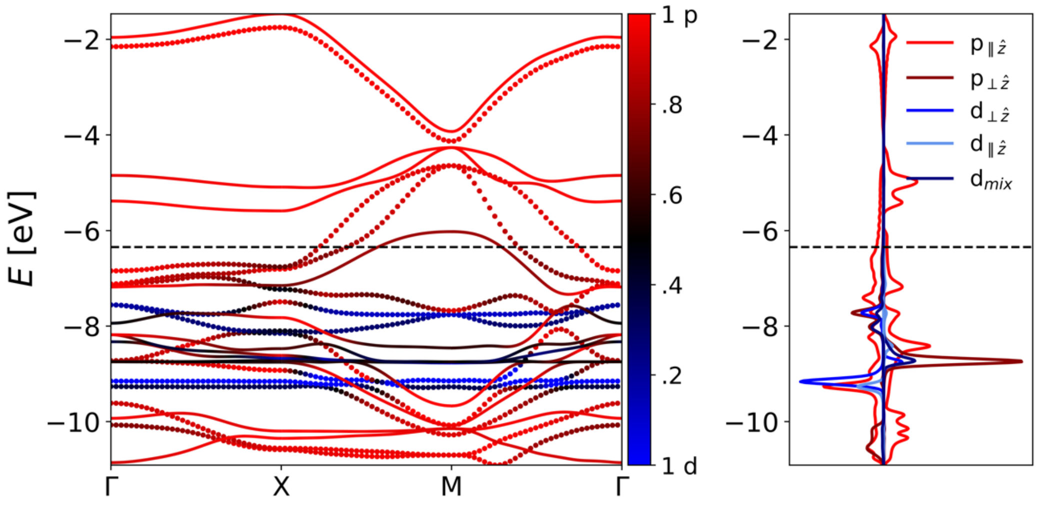

^{5}FeOP

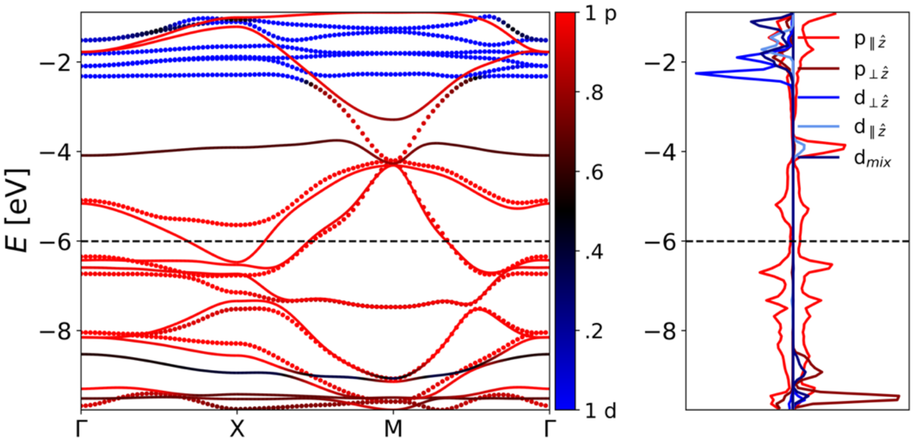

Group $\underline{\mathbf{0}^{t}}$

^{1}CoClP

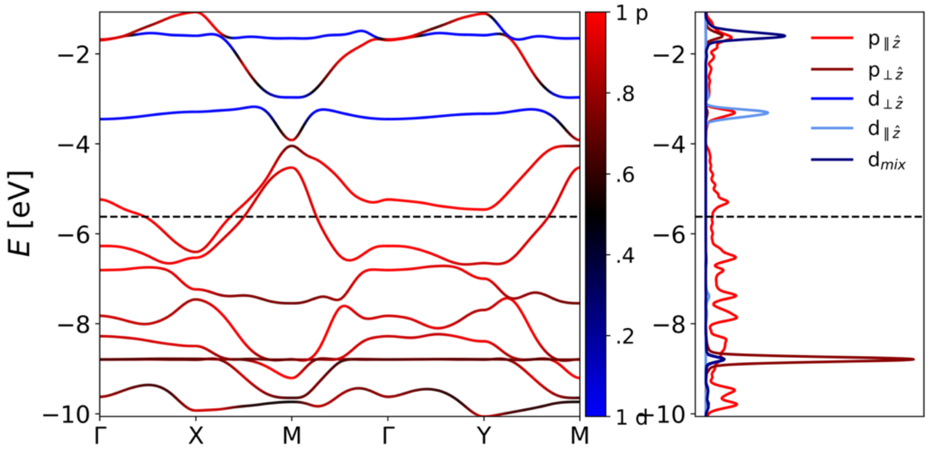

^{1}NiP

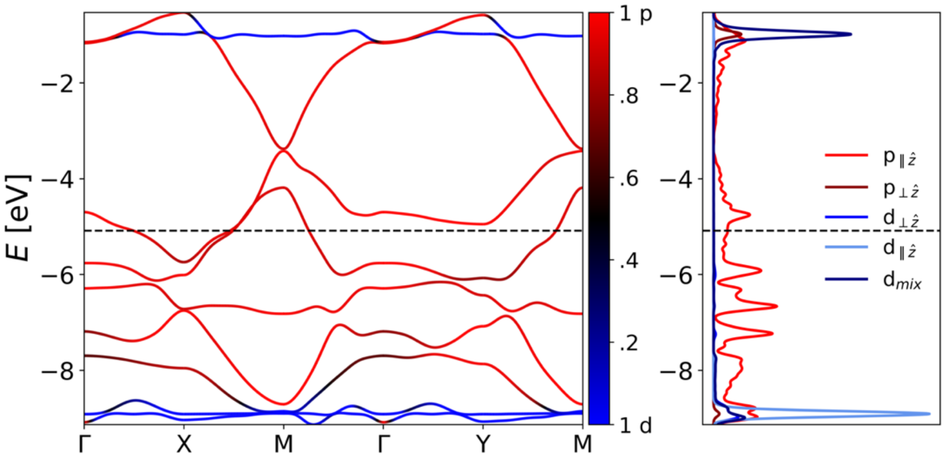

^{2}MnP

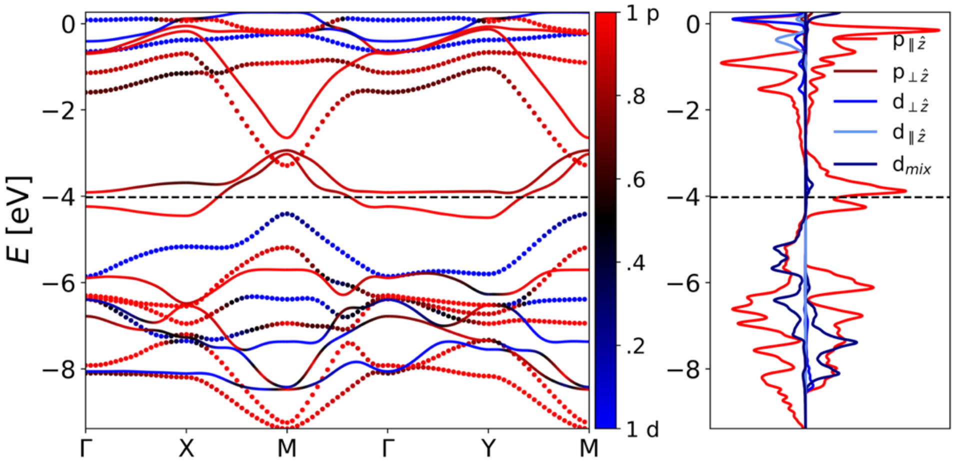

^{6}FeClP

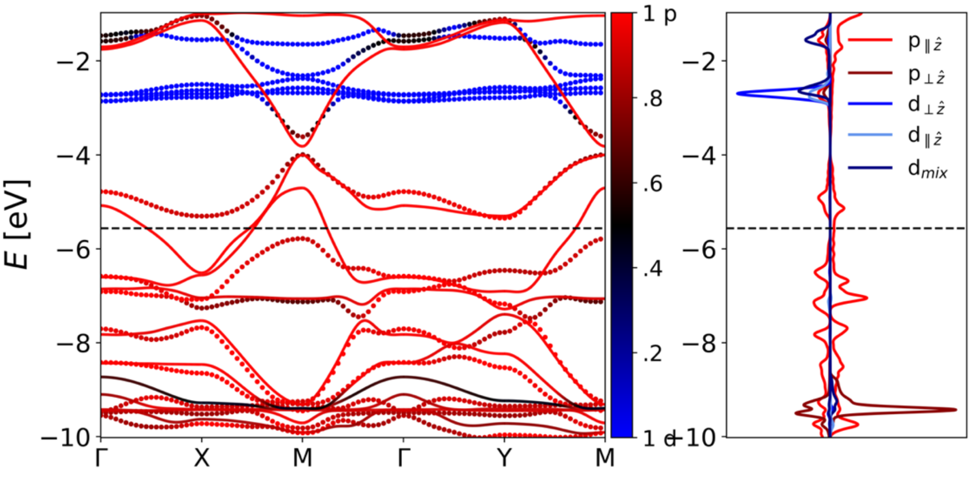

^{2}CoP

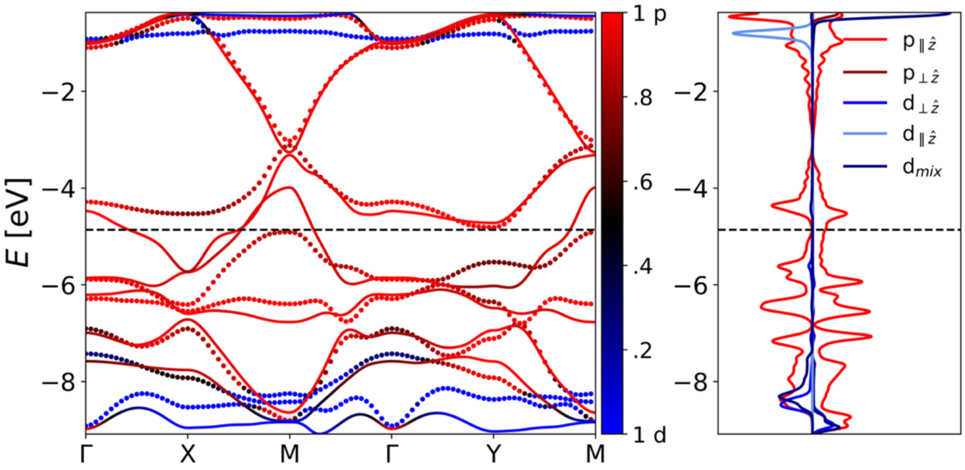

^{5}CoClP

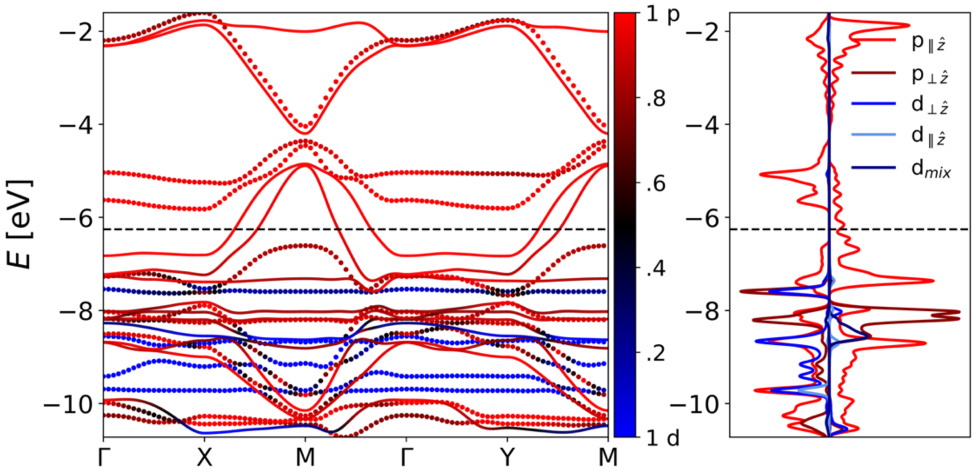

[2]CuP

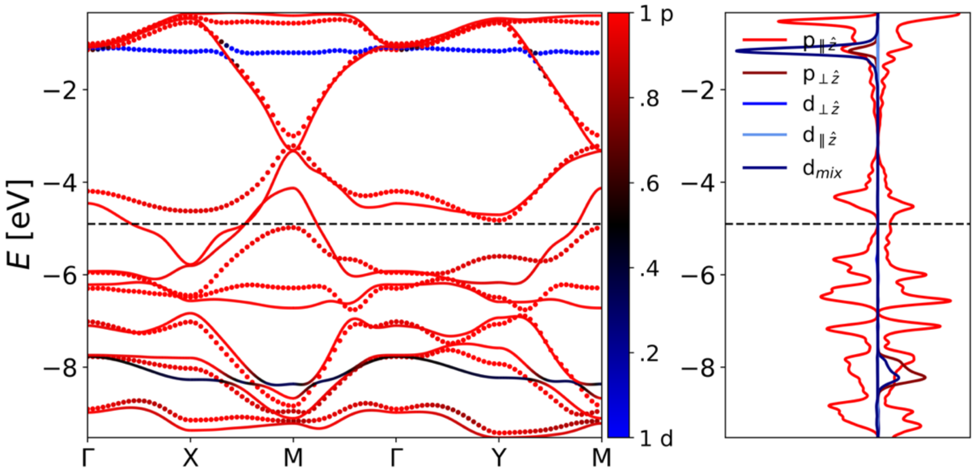

[5]CrP

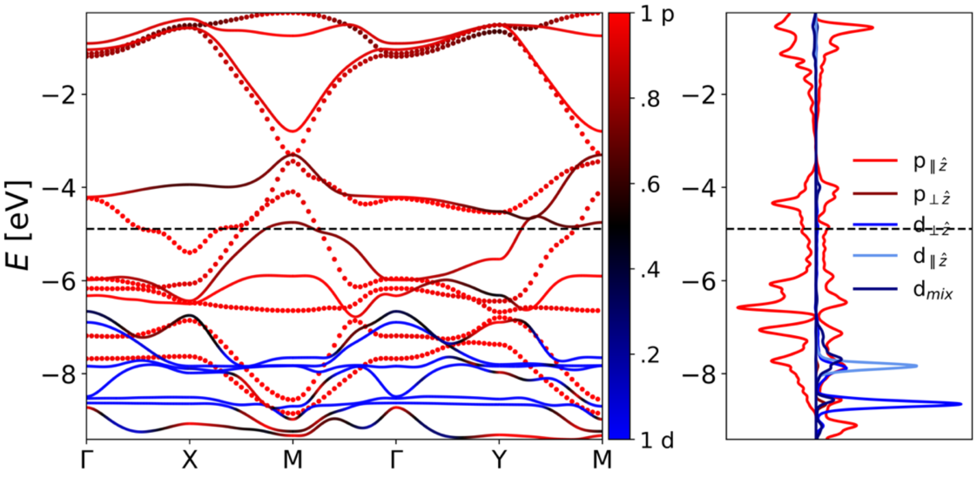

[1]VClP

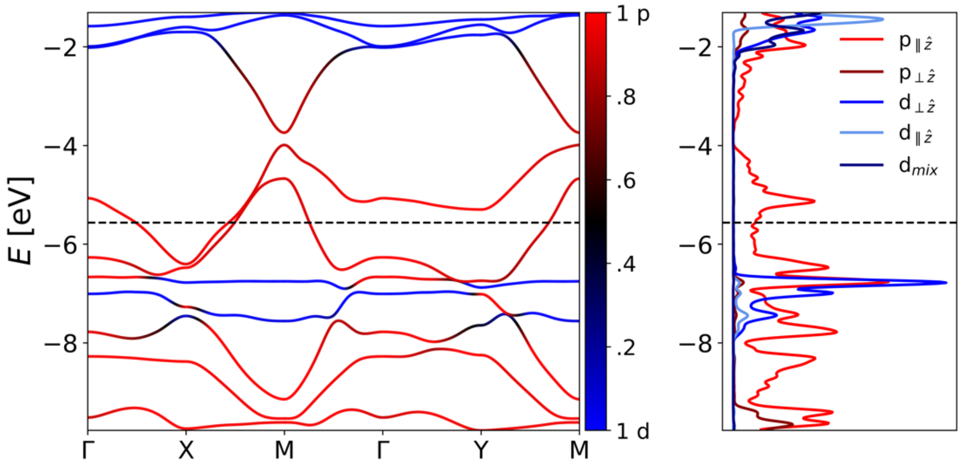

^{3}VClP

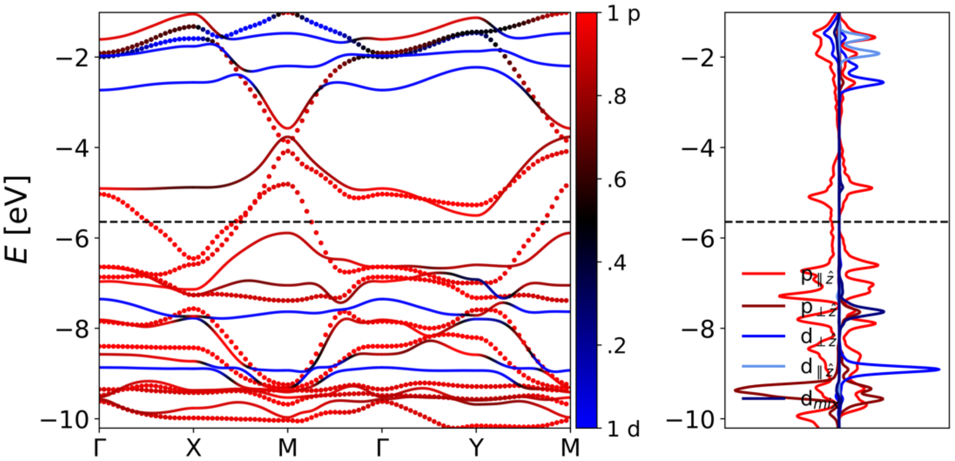

^{1}TiOP

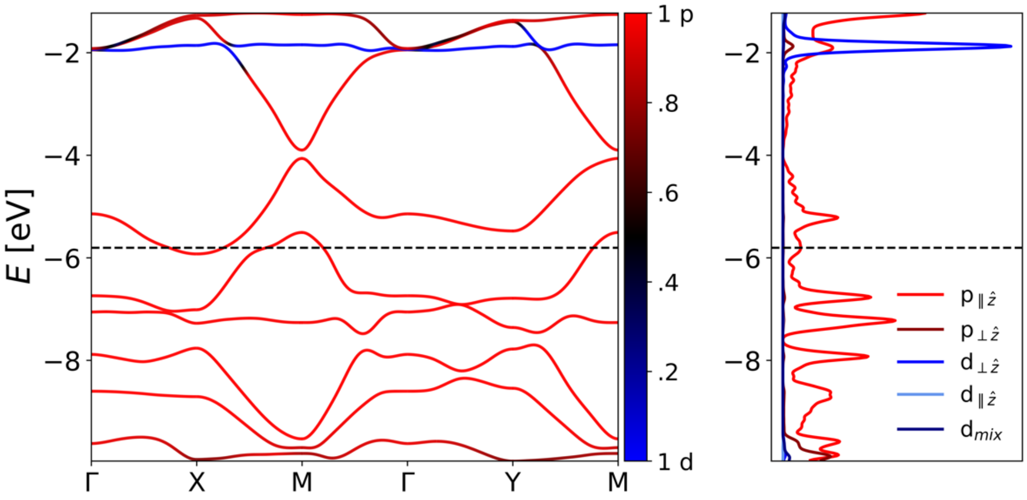

$^{1}FeCl_2P$

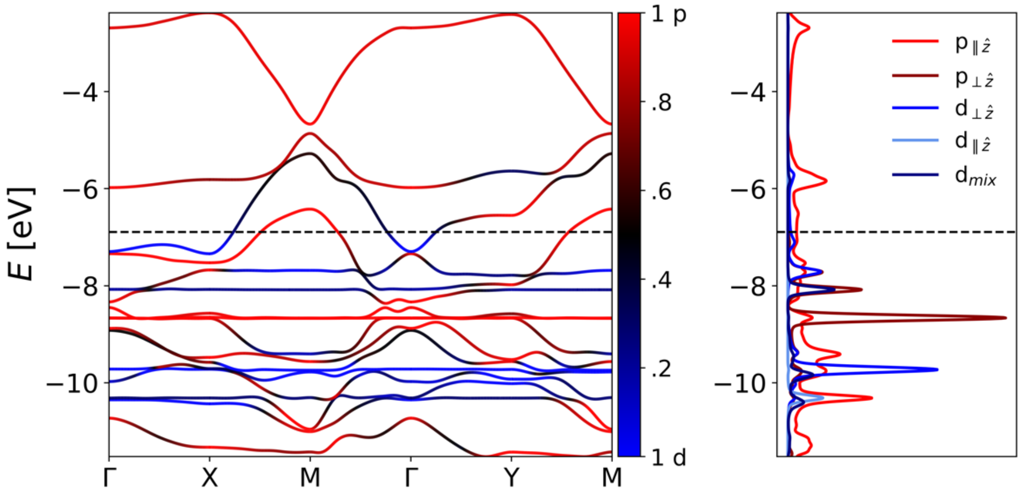

^{1}FeOP

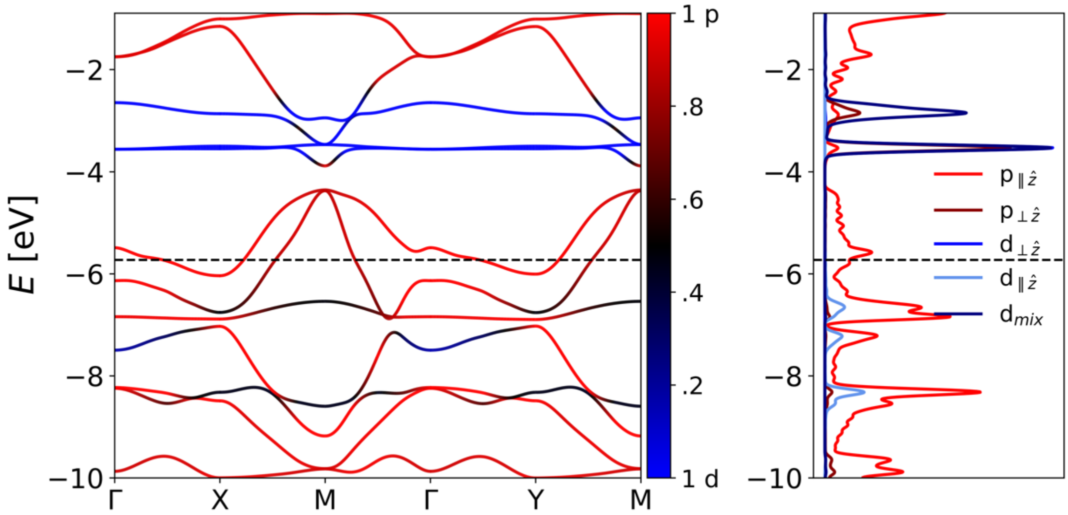

$^{3}NiCl_2P$

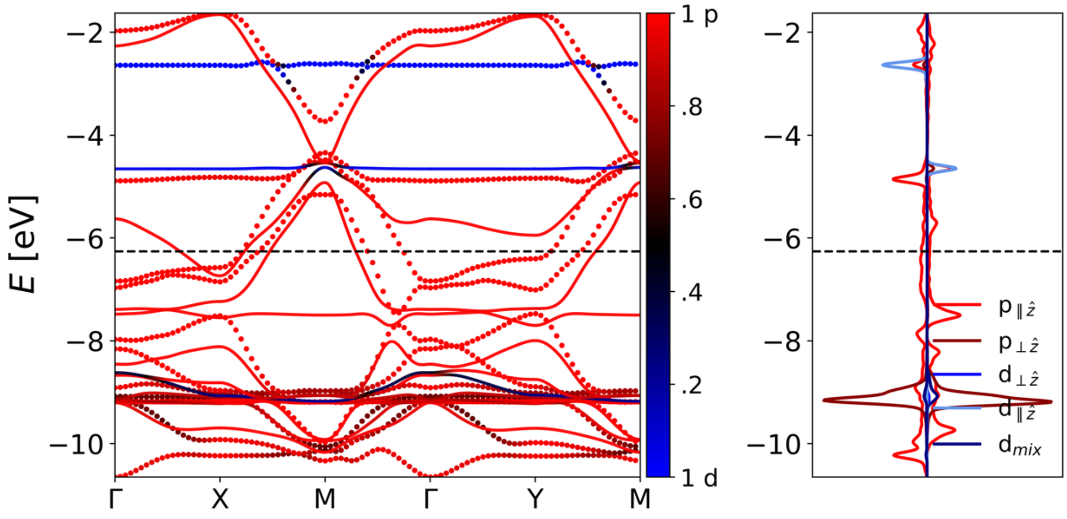

^{2}FeClOP

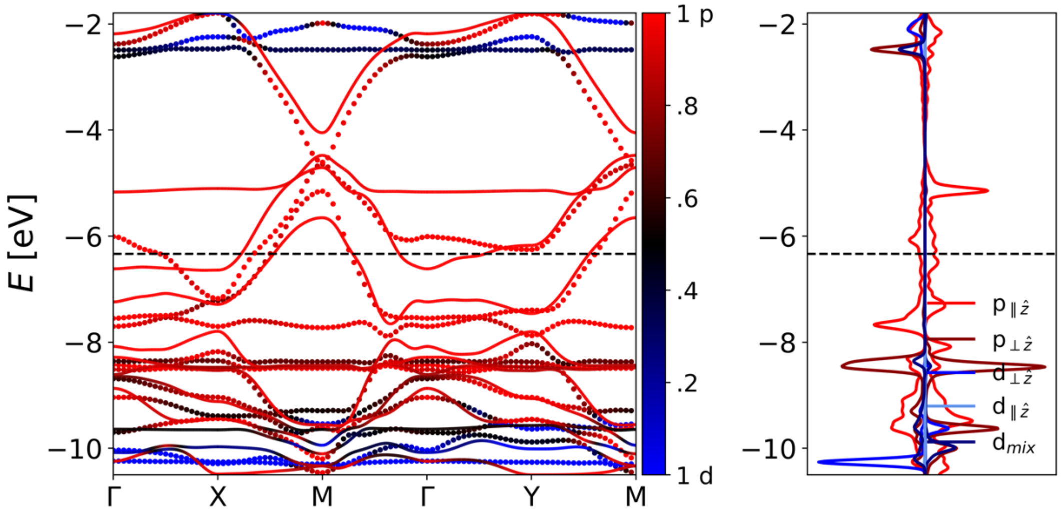

$^{3}FeO_2P$

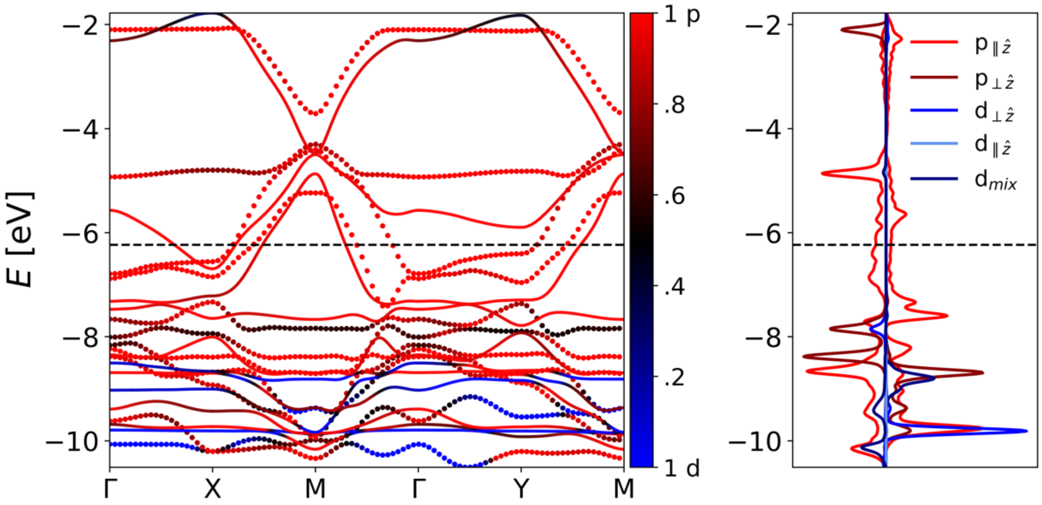

Group **0**^{U}

^{1}MgP

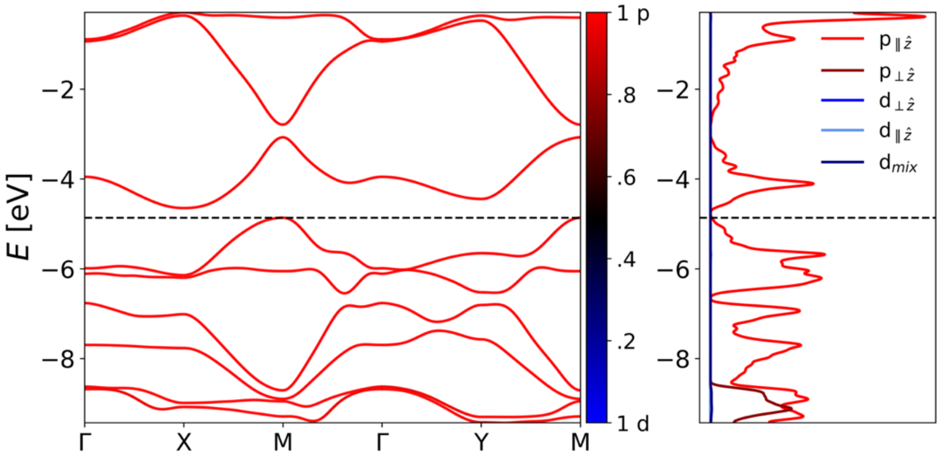

^{1}ScClP

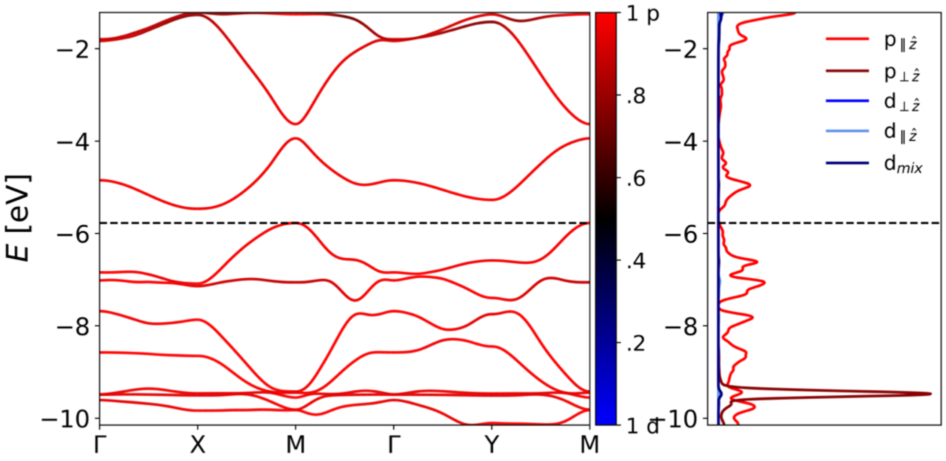

^{1}CrP

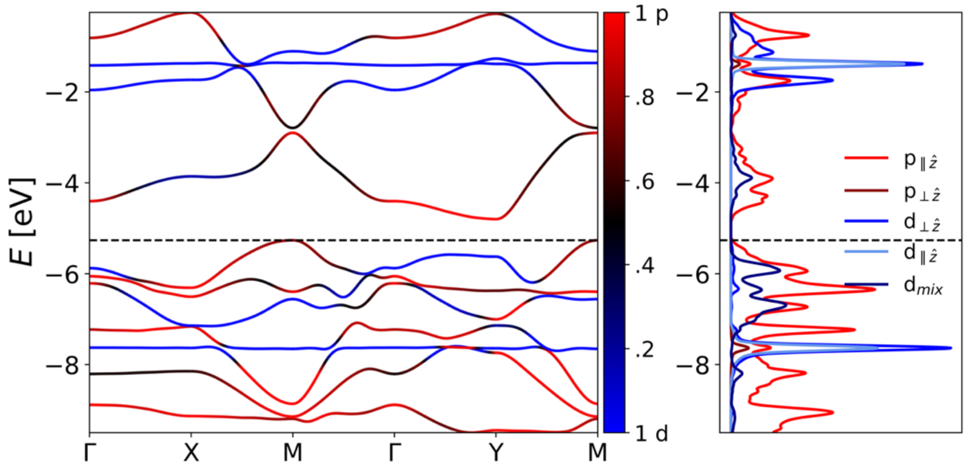

^{1}FeP

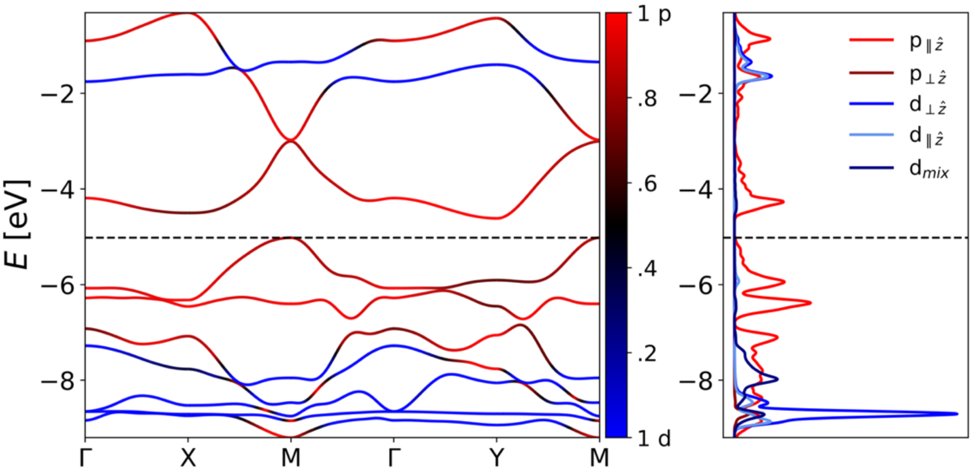

^{5}FeP

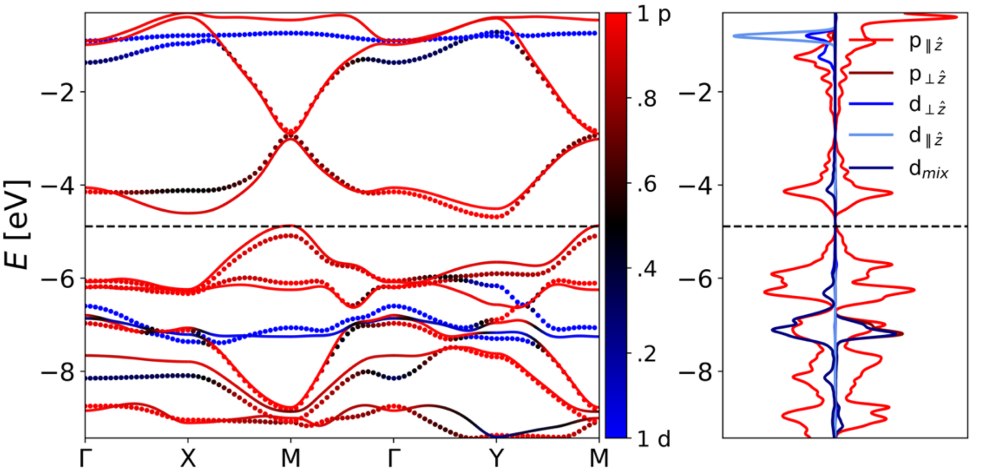

^{1}ZnP

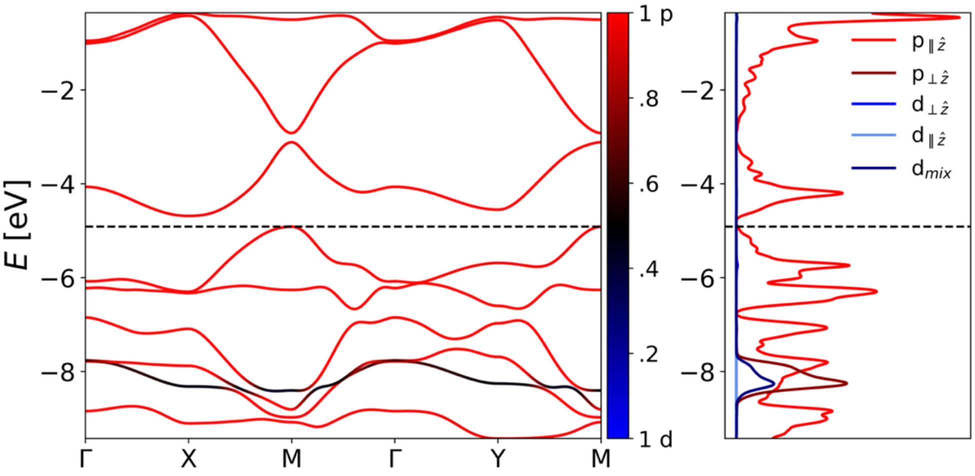

$^{1}FeO_2P$

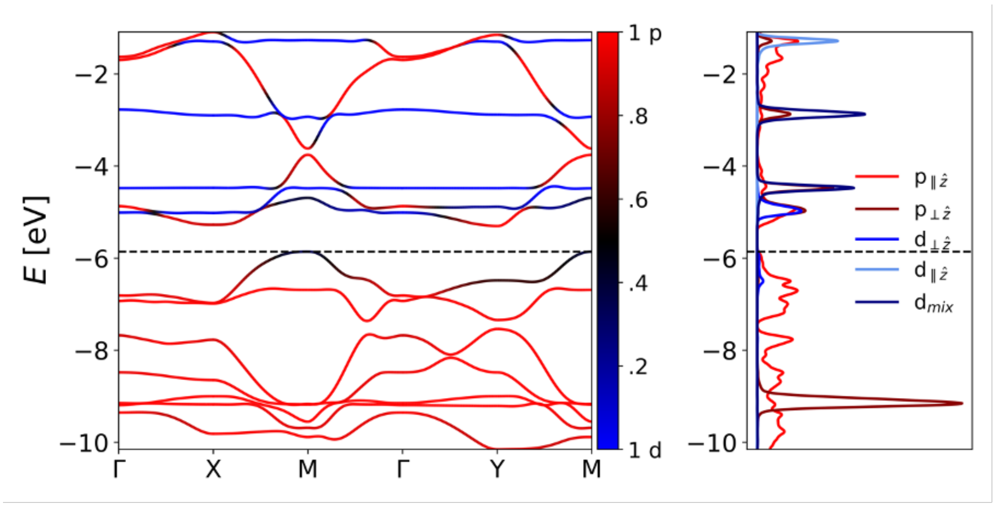

Group $\mathbf{0^{UU}}$

^{2}**CrClP**

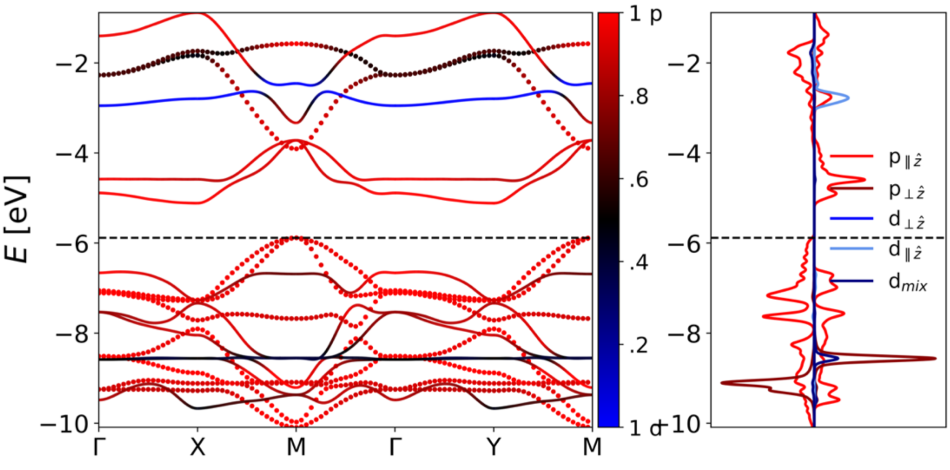

^{3}FeOP

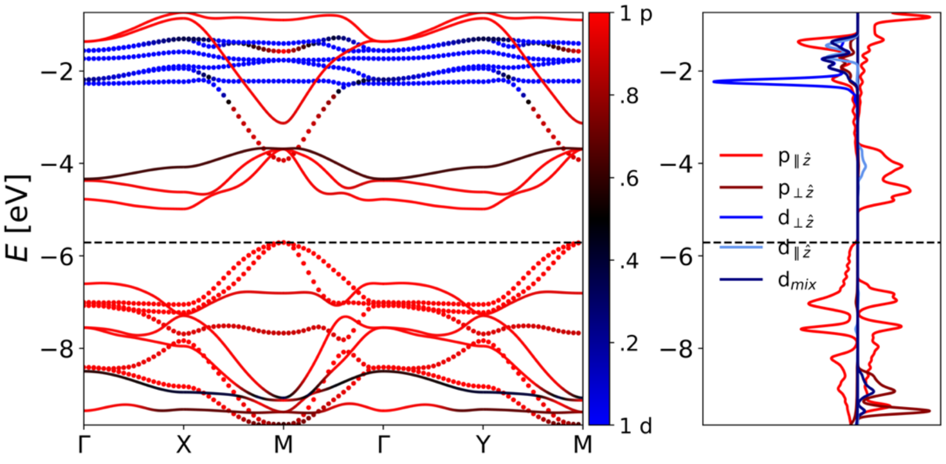

^{2}VP

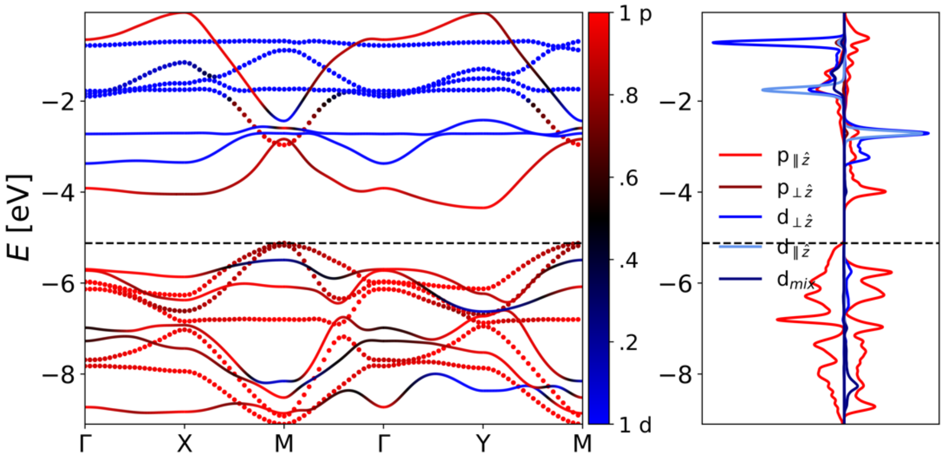

^{3}CoClP

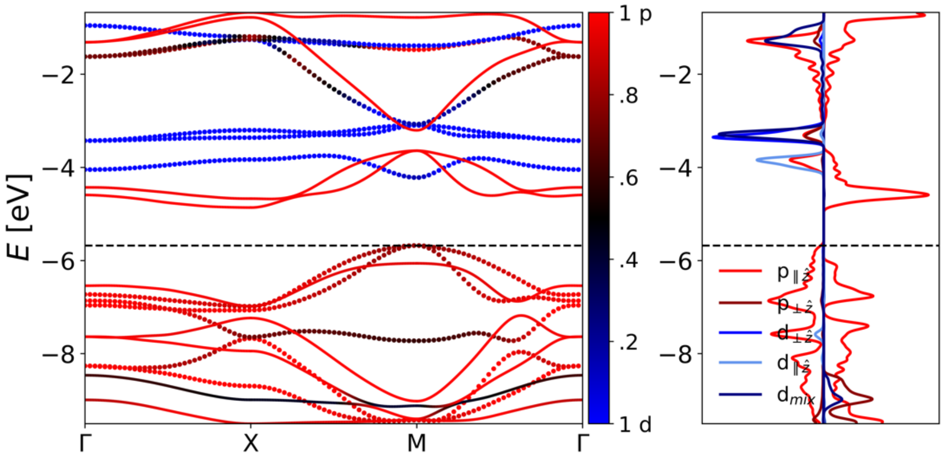

^{3}CrP

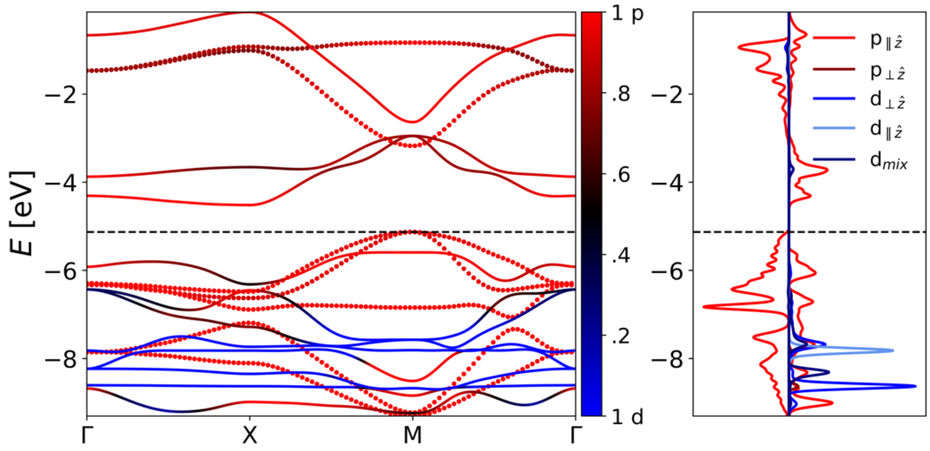

^{2}FeClP

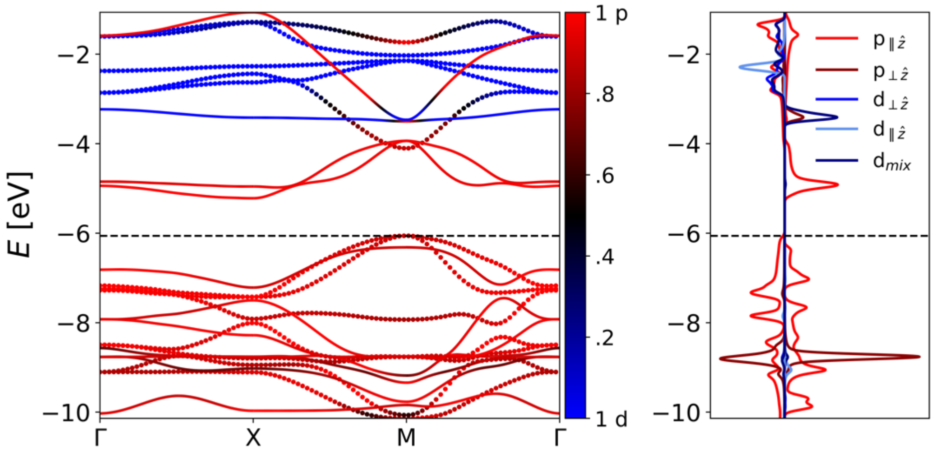

Group **1**

^{2}Sc(III)$^{3+}$P^{3-}

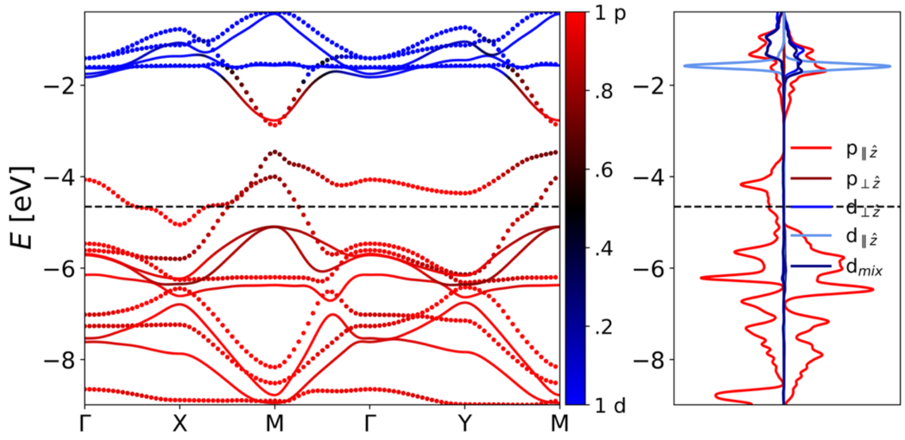

^{4}V(III)$^{3+}$P^{3-}

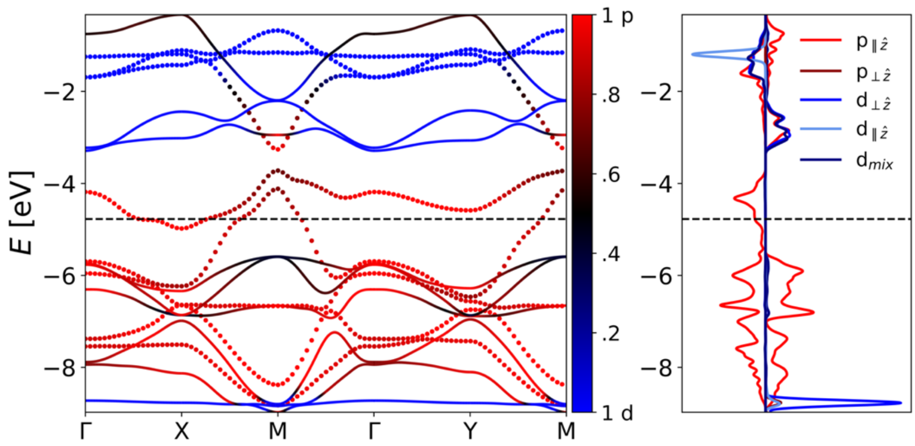

^{3}**Ti(III)$^{3+}$P^{3-}**

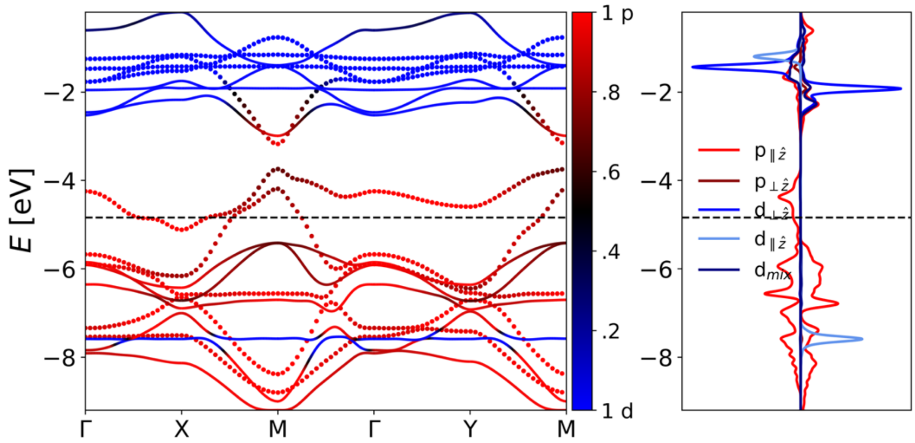

^{2}Ti(III)$^{3+}$P^{3-}

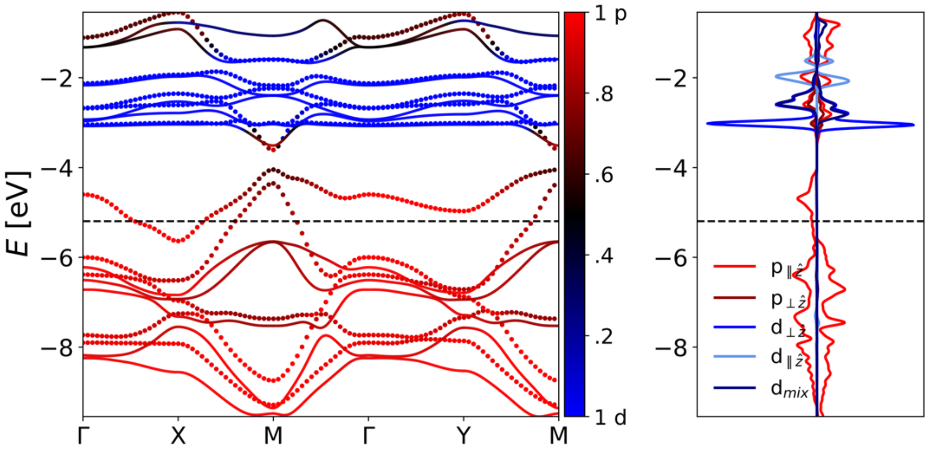

^{4}**Mn(III)$^{3+}$P^{3-}**

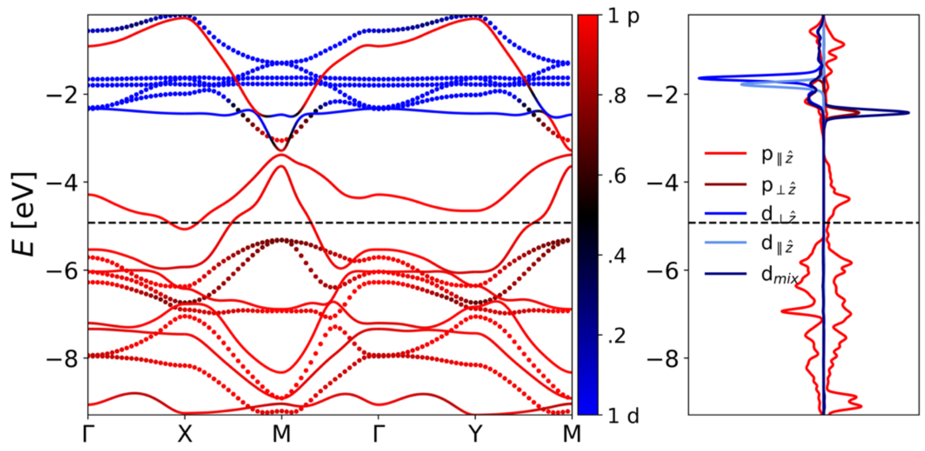

^{6}Mn(III)$^{3+}$P^{3-}

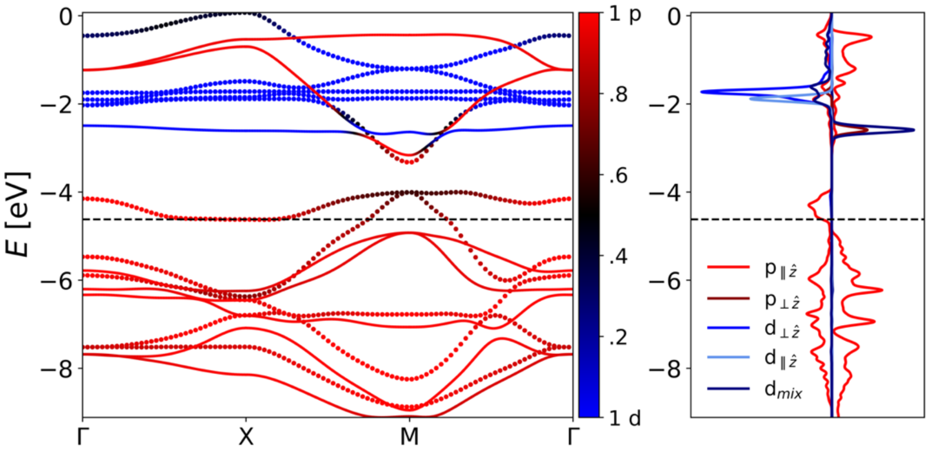

^{3}Fe(III)$^{3+}$P^{3-}

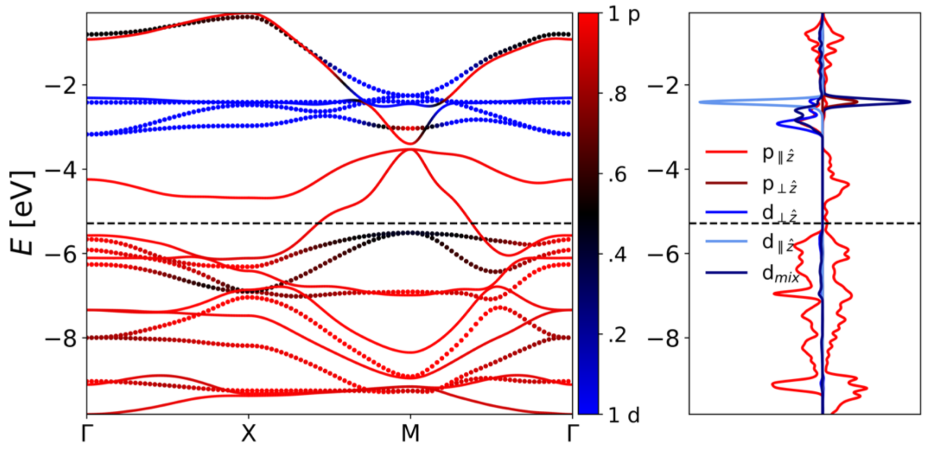

Group **2**

^{1}Ti(IV)$^{4+}$P^{4-}

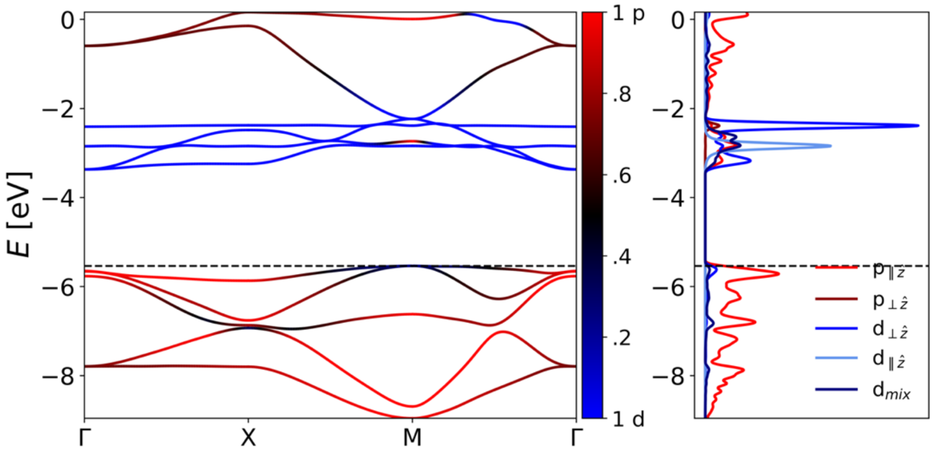

0
−2
−4
−6
−8
E [eV]
Γ
X
M
Γ
1 p
.8
.6
.4
.2
1 d
$p_{\parallel \hat{z}}$
$p_{\perp \hat{z}}$
$d_{\perp \hat{z}}$
$d_{\parallel \hat{z}}$
d_{mix}

References

(1) Shaik, S.; Shurki, A.; Danovich, D.; Hiberty, P. C. A different story of π-delocalizationthe distortivity of π-electrons and its chemical manifestations. *Chemical Reviews* **2001**, *101*, 1501-1540. DOI: 10.1021/cr990363l
(2) Yang, J.; Falletta, S.; Pasquarello, A. Range-separated hybrid functionals for accurate prediction of band gaps of extended systems. *npj Computational Materials* **2023**, *9*, 108. DOI: 10.1038/s41524-023-01064-x
(3) Kaiser, K.; Scriven, L. M.; Schulz, F.; Gawel, P.; Gross, L.; Anderson, H. L. An sp-hybridized molecular carbon allotrope, cyclo[18]carbon. *Science* **2019**, *365*, 1299-1301. DOI: 10.1126/science.aay1914
(4) Karas, L. J.; Jalife, S.; Viesser, R. V.; Soares, J. V.; Haley, M. M.; Wu, J. I. Tetra-tert-butyl-s-indacene is a bond-localized c2h structure and a challenge for computational chemistry. *Angewandte Chemie International Edition* **2023**, *62*, e202307379. DOI: https://doi.org/10.1002/anie.202307379
(5) Casademont-Reig, I.; Guerrero-Avilés, R.; Ramos-Cordoba, E.; Torrent-Sucarrat, M.; Matito, E. How aromatic are molecular nanorings? The case of a six-porphyrin nanoring**. *Angewandte Chemie International Edition* **2021**, *60*, 24080-24088. DOI: https://doi.org/10.1002/anie.202108997
(6) Cohen, A. J.; Mori-Sánchez, P.; Yang, W. Insights into current limitations of density functional theory. *Science* **2008**, *321*, 792-794. DOI: 10.1126/science.1158722
(7) Brunschwig, B. S.; Creutz, C.; Sutin, N. Optical transitions of symmetrical mixed-valence systems in the class ii–iii transition regime. *Chemical Society Reviews* **2002**, *31*, 168-184. DOI: 10.1039/B008034I
(8) Renz, M.; Theilacker, K.; Lambert, C.; Kaupp, M. A reliable quantum-chemical protocol for the characterization of organic mixed-valence compounds. *Journal of the American Chemical Society* **2009**, *131*, 16292-16302. DOI: 10.1021/ja9070859
(9) Parthey, M.; Kaupp, M. Quantum-chemical insights into mixed-valence systems: Within and beyond the robin–day scheme. *Chemical Society Reviews* **2014**, *43*, 5067-5088. DOI: 10.1039/C3CS60481K
(10) Renz, M.; Kaupp, M. Predicting the localized/delocalized character of mixed-valence diquinone radical anions. Toward the right answer for the right reason. *The Journal of Physical Chemistry A* **2012**, *116*, 10629-10637. DOI: 10.1021/jp308294r
(11) Kröncke, S.; Herrmann, C. Toward a first-principles evaluation of transport mechanisms in molecular wires. *Journal of Chemical Theory and Computation* **2020**, *16*, 6267-6279. DOI: 10.1021/acs.jctc.0c00667
(12) Herrmann, C. Electronic communication as a transferable property of molecular bridges? *The Journal of Physical Chemistry A* **2019**, *123*, 10205-10223. DOI: 10.1021/acs.jpca.9b05618
(13) Tanaka, Y.; Takahashi, H.; Akita, M. Estimation of electron distribution over dinuclear organometallic molecular wires by “ir tag” analysis of ancillary acyl-cp ligands. *ACS Organic & Inorganic Au* **2022**, *2*, 327-342. DOI: 10.1021/acsorginorgau.2c00005
(14) Magnera, T. F.; Dron, P. I.; Bozzone, J. P.; Jovanovic, M.; Rončević, I.; Tortorici, E.; Bu, W.; Miller, E. M.; Rogers, C. T.; Michl, J. Porphene and porphite as porphyrin analogs of graphene and graphite. *Nature Communications* **2023**, *14*, 6308. DOI: 10.1038/s41467-023-41461-w
